# Long-term evolution of the burden of HIV/AIDS in older adults: a global burden of disease analysis in individuals aged ≥75 years in G7 countries

**DOI:** 10.3389/fpubh.2026.1811915

**Published:** 2026-05-11

**Authors:** Ruizhuang Sun, Yan Jiang, Yingyu Yan, Tingxia Zhou, Chonghui Xu, Jingting Wang, Long Ye, Jun Meng

**Affiliations:** 1 Department of Laboratory Medicine, Ruijin-Hainan Hospital, Shanghai Jiaotong University School of Medicine (Hainan Boao Research Hospital), Qionghai, Hainan, China; 2 Shenyang Medical College, Shenyang, Liaoning, China; 3 Department of Nursing, Ruijin Hospital, Shanghai Jiaotong University School of Medicine, Shanghai, China; 4 Department of Nursing, Ruijin-Hainan Hospital, Shanghai Jiaotong University School of Medicine (Hainan Boao Research Hospital), Qionghai, Hainan, China; 5 Department of Laboratory Medicine, Boao International Hospital of Shanghai University of Traditional Chinese Medicine, Qionghai, Hainan, China; 6 Hainan Vocational University of Science and Technology, Haikou, Hainan, China; 7 Department of Laboratory Medicine, Ruijin Hospital, Shanghai Jiaotong University School of Medicine, Shanghai, China

**Keywords:** antiretroviral therapy, Disability-adjusted life years, epidemiological transition, G7 countries, HIV/AIDS, older adults

## Abstract

**Background:**

With the widespread implementation of antiretroviral therapy (ART), human immunodeficiency virus/acquired immunodeficiency syndrome (HIV/AIDS) has gradually transitioned from a highly fatal infectious disease to a manageable chronic condition. Simultaneously, global population aging has accelerated, making older adults with HIV (≥75 years) an increasingly important group in terms of disease burden. However, the epidemiological characteristics, disease spectrum, and long-term trends of this population—including key metrics such as incidence, mortality, prevalence, and disability-adjusted life years (DALYs)—remain inadequately assessed, particularly regarding epidemiological transitions and inter-country differences in this age group across G7 nations.

**Methods:**

Data on HIV/AIDS and its subtypes from 1990 to 2021 were obtained from the Global Burden of Disease (GBD) 2021 database for G7 countries (Canada, France, Germany, Italy, Japan, the United Kingdom, and the United States) among individuals aged ≥75 years. Metrics included age-standardized incidence rate (ASIR), age-standardized mortality rate (ASMR), age-standardized prevalence rate (ASPR), and DALYs. Stratified analyses were performed by age groups (75–79, 80–84, 85–89, 90–94, ≥95 years), sex, and disease subtype. Long-term trends were assessed using Joinpoint regression, calculating the annual percent change (APC) and average annual percent change (AAPC).

**Results:**

Between 1990 and 2021, the incidence and mortality of HIV/AIDS in older adults declined continuously in most G7 countries, reflecting the effectiveness of ART and public health interventions. In contrast, prevalence and DALYs increased in some countries, indicating an asymmetric pattern of “decreasing incidence and mortality, but increasing survival and disability.” Considerable heterogeneity existed among countries: the United States and several European countries exhibited a high burden among surviving individuals, whereas Japan showed a consistently increasing trend. Sex-specific analysis revealed a higher overall burden in males than females, although the sex gap narrowed in the oldest age groups. The disease spectrum shifted over time, with a declining burden of drug-susceptible tuberculosis and non-communicable chronic diseases (conditions associated with HIV/AIDS) emerging as the primary drivers of DALYs.

**Conclusion:**

Older adults should be incorporated into HIV prevention, screening, and health management strategies, with particular attention to chronic disease management, functional maintenance, and multidisciplinary care to address the long-term health challenges posed by aging HIV cohorts.

## 1 Introduction

Over the past four decades, the widespread implementation of ART has significantly improved survival outcomes for patients with HIV/AIDS (1, 2), transforming this condition from a highly fatal infectious disease into a manageable chronic illness. Concurrently, global population aging has accelerated (3), reshaping the age structure of people living with HIV, with an increasing proportion surviving to advanced ages. Previous research has mainly focused on younger populations, while the epidemiological characteristics, disease spectrum, and long-term trends of HIV among older adults remain underexplored.

The older adult HIV/AIDS population exhibits distinct characteristics with respect to disease burden. On one hand, this population frequently presents with multiple comorbidities, and the interaction between immunosenescence (4, 5) and chronic infection may alter disease progression pathways and mortality risk. On the other hand, long-term ART use has substantially extended survival, leading to continuous accumulation of prevalence and disability burden. Furthermore, trends in HIV/AIDS-associated tuberculosis and drug-resistant tuberculosis among older adults may differ from those observed in the general population (6).

In recent years, studies on HIV/AIDS disease burden based on the GBD framework have proliferated, deepening our understanding of the epidemiological characteristics of this disease at global, regional, and population-specific levels. For instance, some studies have systematically assessed trends in HIV/AIDS incidence, mortality, and disability-adjusted life years (DALYs) from 1990 to 2021 globally and across regions, projecting continued overall burden reduction through 2030 and providing crucial evidence for global control strategies (7). Additionally, research on HIV/AIDS-associated tuberculosis has further elucidated long-term trends of different drug-resistant tuberculosis types globally, highlighting the ongoing public health challenge of rising drug-resistant tuberculosis in certain regions (8). From a life-course perspective, studies have also examined trends in HIV and other sexually transmitted infections among adolescents and young adults (10–24 years) (9) as well as older adults (60–89 years), revealing significant disease burden disparities across age groups.

Nevertheless, several limitations remain. First, most studies focus on all-age populations; although age stratification is applied, independent and systematic characterization of older adults is limited, particularly for those at advanced ages (7). Second, studies targeting older adult populations typically define age ranges broadly (e.g., 60–89 years) and often focus on incidence and DALYs, without integrating multiple indicators such as incidence, mortality, and prevalence, nor adequately examining subtype-specific patterns such as drug-resistant tuberculosis (8). Third, while some studies have projected future HIV burden, these analyses primarily emphasize low- and middle-income regions, with limited attention to aging populations in high-income countries (7).

Therefore, despite the global decline in HIV/AIDS burden, it remains unclear whether older adults are developing disease burden patterns that differ from traditional epidemiological trends due to a lack of long-term, standardized evidence. Specifically, in high-income settings such as the G7 countries, whether HIV/AIDS is shifting from an infection- and mortality-dominated pattern toward one characterized by long-term survival and disability burden, and how associated conditions such as tuberculosis evolve with age, remain insufficiently understood.

Accordingly, this study focuses on individuals aged ≥75 years in G7 countries, systematically evaluating the epidemiological characteristics and long-term trends of HIV/AIDS and its related subtypes from 1990 to 2021. As countries with advanced healthcare systems and pronounced population aging, G7 nations provide a valuable context for comparative analysis. Despite progress in HIV prevention and treatment, whether the burden among older adults continues to decline, whether cross-country heterogeneity exists, and whether disease burden is shifting toward chronic disability remain unclear.

Utilizing the GBD database (10), this study systematically evaluates the epidemiological characteristics of HIV/AIDS and its related subtypes among individuals aged ≥75 years in G7 countries from 1990 to 2021. By analyzing long-term trends in age-standardized incidence rate (ASIR), age-standardized mortality rate (ASMR), age-standardized prevalence rate (ASPR), and DALYs, combined with sex- and subtype-stratified analyses and Joinpoint regression, this study aims to characterize evolving burden patterns of HIV/AIDS in older adults and cross-country differences, providing evidence to inform targeted HIV control strategies and resource allocation for aging populations.

## 2 Methods

### 2.1 Data sources and processing

To systematically evaluate the disease burden of HIV/AIDS and its related subtypes among individuals aged ≥75 years in G7 countries (Canada, France, Germany, Italy, Japan, the United Kingdom, and the United States), this study obtained the latest epidemiological data through the GBD 2021 Results Tool. The GBD study integrates multi-source health data from over 200 countries and territories worldwide and is recognized as one of the authoritative databases for quantifying disease burden and health loss internationally.

Disease categories included in this study comprised overall HIV/AIDS and its related subtypes, specifically: HIV/AIDS, HIV/AIDS with drug-susceptible tuberculosis, HIV/AIDS with multidrug-resistant tuberculosis without extensive drug resistance, HIV/AIDS with extensively drug-resistant tuberculosis, and other diseases resulting from HIV/AIDS. For these disease categories, data on incident cases, deaths, DALYs, and corresponding ASIR, ASMR, and ASPR from 1990 to 2021 were extracted for each country. All indicators were age-standardized according to the GBD standard population.

### 2.2 Age and sex stratified analysis

Given that HIV/AIDS burden among older adults may exhibit significant age and sex differences, this study further conducted age and sex stratified analyses. The study population was restricted to individuals aged ≥75 years and divided into five age groups at 5-year intervals: 75–79, 80–84, 85–89, 90–94, and≥95 years.

On this basis, the distribution characteristics of HIV/AIDS case numbers and DALYs were compared across different age groups and between sexes (male and female) to reveal the age structure characteristics and sex differences in HIV/AIDS burden among the older adult population.

### 2.3 Joinpoint regression analysis

To assess temporal trends in age-standardized HIV/AIDS indicators from 1990 to 2021, this study employed Joinpoint regression analysis to model ASIR and ASMR. Joinpoint regression can partition long-term trends into several statistically significant phases of change, facilitating identification of periods of increase, plateau, and decline.

The model was configured to allow a maximum of two joinpoints (i.e., up to three time segments), with optimal model selection through stepwise fitting. The APC and its 95% confidence interval (95% CI) were used to evaluate the direction and magnitude of trend changes within each time segment. Additionally, the AAPC was calculated to describe the overall long-term trends in ASIR and ASMR from 1990 to 2021.

### 2.4 Statistical analysis

All statistical analyses were performed using R software (version 4.2.1, R Foundation for Statistical Computing, Vienna, Austria). When both APC and its 95% CI were greater than zero, the indicator was determined to show a significant increasing trend during the corresponding period; when both were less than zero, a significant decreasing trend was determined; if the 95% CI included zero, the trend change during that period was considered not statistically significant.

When the *p*-value for APC or AAPC was less than 0.05, the corresponding trend was considered statistically significant. All tests were two-sided, with the significance level set at 0.05.

## 3 Results

### 3.1 National heterogeneity, sex reversal gradient, and disease spectrum differentiation in HIV/AIDS burden among individuals aged ≥75 years in G7 countries

**Figure 1:**
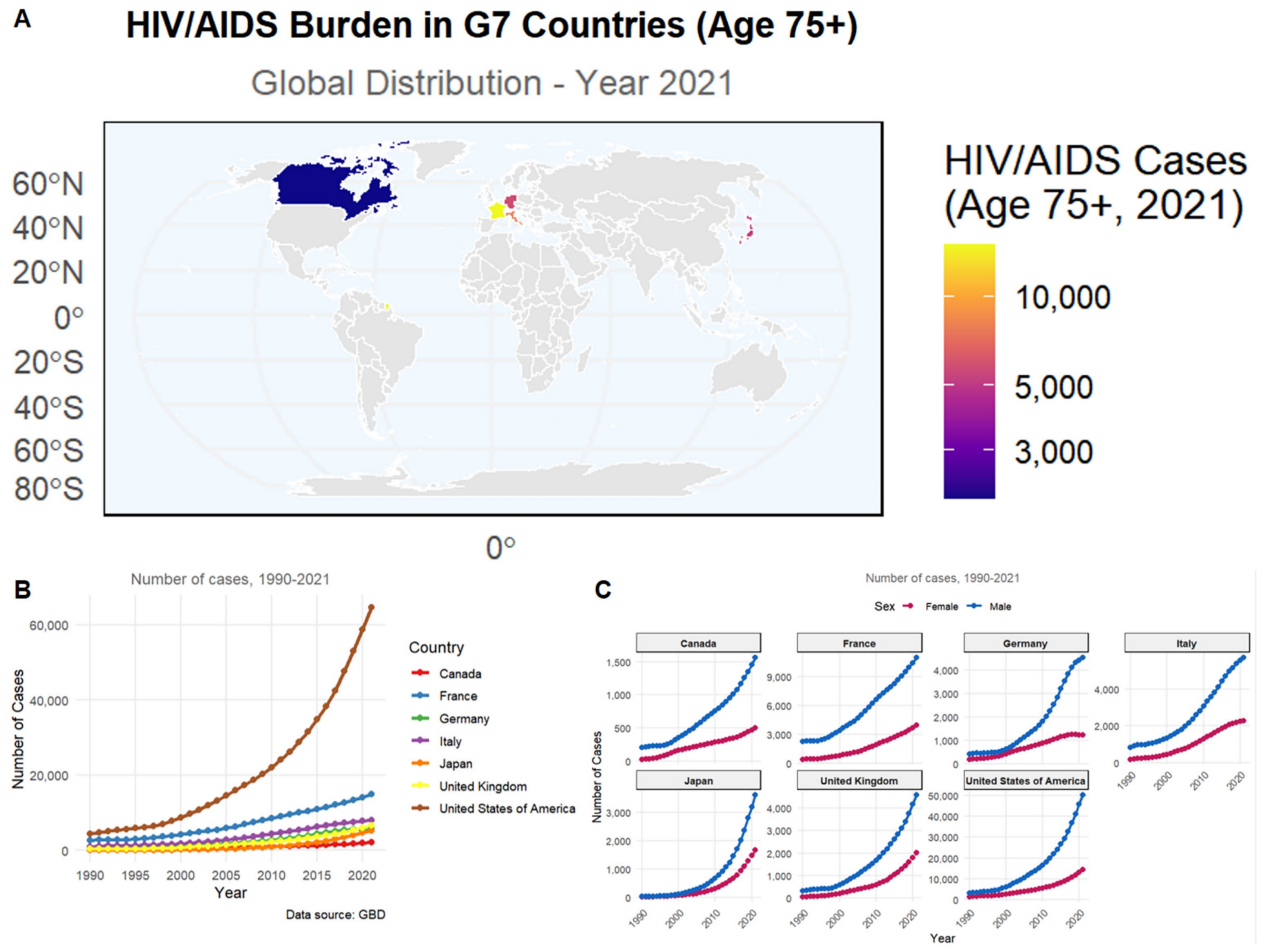
HIV/AIDS burden among adults aged ≥75 years in G7 countries. **(A)** Global map showing the distribution of HIV/AIDS cases in 2021, with color intensity indicating the number of cases. **(B)** Temporal trends in the number of cases from 1990 to 2021 for each G7 country (Canada, France, Germany, Italy, Japan, the United Kingdom, and the United States). **(C)** Sex-stratified trends (female vs. male) in HIV/AIDS cases from 1990 to 2021 for each country. Data source: Global Burden of Disease (GBD).

**Figure 2:**
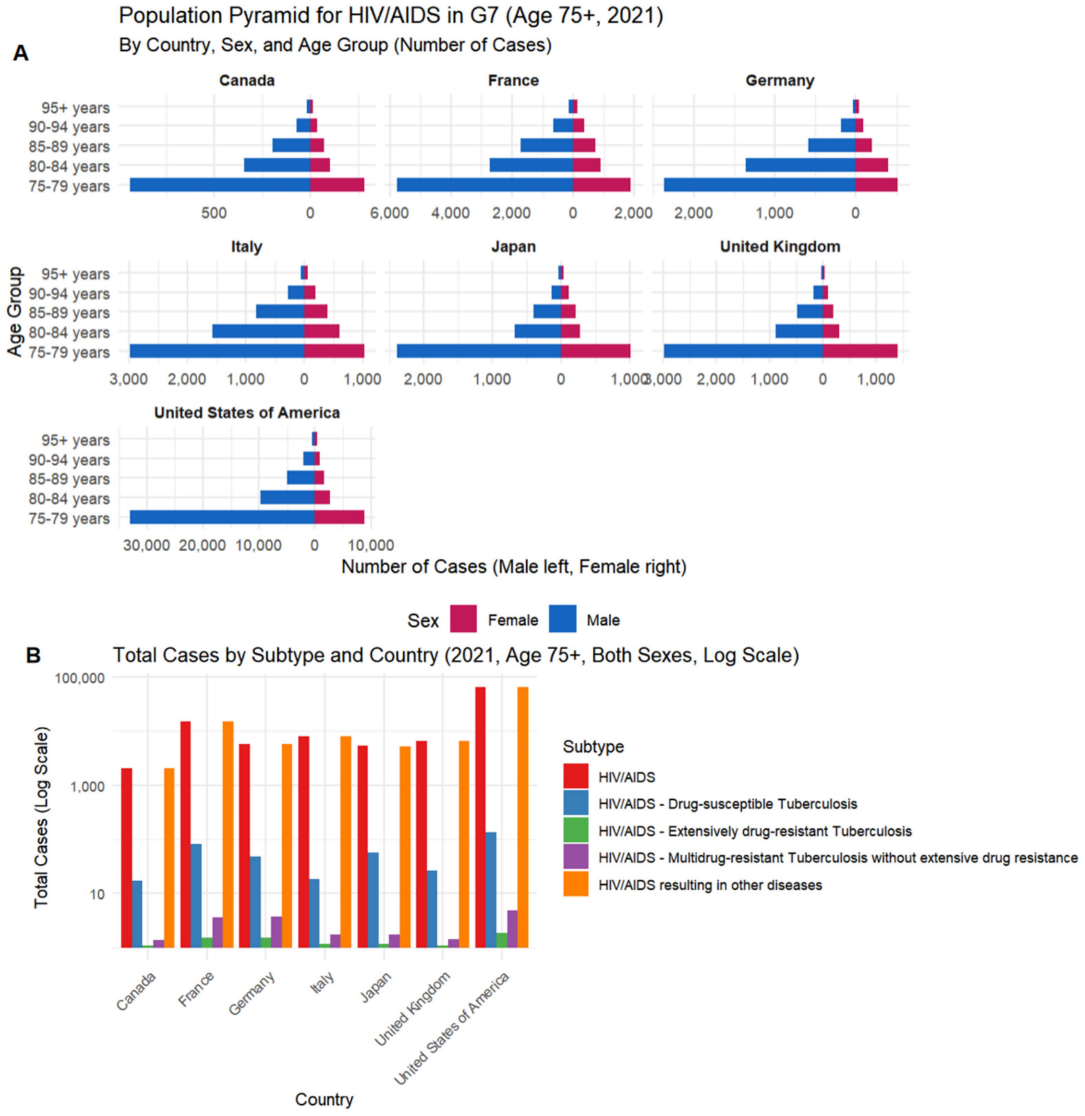
Age–sex structure and subtype composition of HIV/AIDS burden in G7 countries among adults aged ≥75 years in 2021. **(A)** Population pyramids showing the number of HIV/AIDS cases by age group (75–79 to ≥95 years) and sex for each G7 country; males are displayed on the left and females on the right. **(B)** Total cases by HIV/AIDS subtype across G7 countries in 2021 (both sexes), presented on a logarithmic scale, including HIV/AIDS alone, HIV/AIDS with drug-susceptible tuberculosis, HIV/AIDS with multidrug-resistant tuberculosis (with or without extensive drug resistance), HIV/AIDS with extensively drug-resistant tuberculosis, and HIV/AIDS resulting in other diseases. Data source: Global Burden of Disease (GBD).

Among individuals aged ≥75 years, HIV/AIDS burden in G7 countries did not simply follow a linear distribution according to economic scale but exhibited marked national heterogeneity. Although case numbers in the United States far exceeded those in other countries, European countries (France, Germany, Italy) showed smaller declines in case numbers in the oldest age groups (≥85 years), demonstrating a more pronounced “aging tail” distribution pattern, while Japan and Canada exhibited rapid case attrition after age 80 (Figures 1A, 2A). This phenomenon suggests significant differences in cumulative survival effects among older adult HIV populations across countries, rather than being determined solely by overall case volume.

Regarding sex structure, most countries showed clear male predominance in the 75–79 age group, but the sex gap gradually narrowed with advancing age, with some countries showing a relative increase in the proportion of female cases in the ≥90 age group, particularly evident in France, Italy, and Germany (Figure 2A). This “convergence of sex ratios at advanced age” contrasts with the sex pattern of HIV epidemics in young and middle-aged populations, suggesting that female HIV-infected individuals may have longer survival accumulation or different mortality selection at very advanced ages.

**Figure 3:**
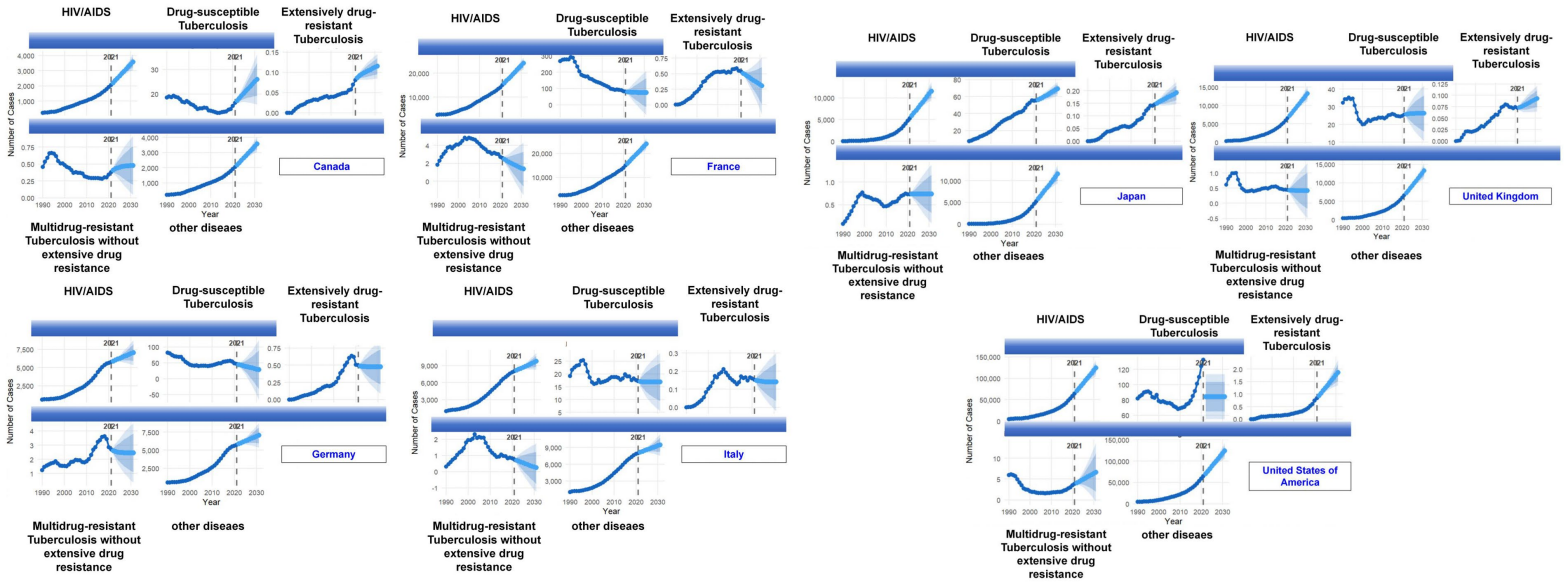
Trends in HIV/AIDS cases and HIV/TB-related subtypes among adults aged ≥75 years in G7 countries, 1990–2030. For each country (Canada, France, Germany, Italy, Japan, the United Kingdom, and the United States), panels show time-series of the number of cases for overall HIV/AIDS and selected HIV-associated outcomes, including HIV/AIDS with drug-susceptible tuberculosis, HIV/AIDS with multidrug-resistant tuberculosis (without extensive drug resistance), HIV/AIDS with extensively drug-resistant tuberculosis, and HIV/AIDS resulting in other diseases. The dashed vertical line marks year 2021; shaded bands indicate projected uncertainty through 2030.

In terms of disease composition, HIV/AIDS burden across countries was not dominated by tuberculosis-related subtypes but primarily comprised “HIV/AIDS alone” and “other diseases resulting from HIV/AIDS,” a structure highly consistent across nations (Figure 2B). Notably, although the United States had the highest absolute case numbers across all subtypes, the proportion of drug-resistant tuberculosis-related subtypes was not significantly higher than in European countries, indicating that the primary health burden among older adult HIV populations is gradually shifting from classic opportunistic infections to multisystem chronic comorbidities (Figures 2B, 3).

Long-term trend and projection results further demonstrated that from 1990 to 2021, the rate of HIV/AIDS case increase across countries was not synchronized with tuberculosis-related subtypes. Overall HIV/AIDS cases showed accelerated growth, while multidrug-resistant and extensively drug-resistant tuberculosis cases grew slowly, with some countries even reaching a plateau (Figure 3). Conversely, other diseases resulting from HIV/AIDS continued to rise in all G7 countries and became the main driver of growth during the projection period, particularly prominent in the United States, the United Kingdom, and Germany (Figure 3). This divergent trend indicates that HIV/AIDS burden in older adults is transitioning from an “infection-dominated” pattern to an “aging-related comorbidity-dominated” pattern.

### 3.2 Age-standardized incidence rate: national divergence within overall decline and the Japanese exception

From 1990 to 2010, overall HIV/AIDS incidence among individuals aged ≥75 years showed declining trends in most G7 countries, suggesting the effectiveness of early prevention and treatment strategies. For example, the ASIR in the United States decreased from 26.44 to 17.50, with AAPC of −1.43% (95% CI: −2.46 to −0.39%); Italy exhibited a similar declining pattern. In contrast, Japan showed a marked increase during the same period, with ASIR rising from 0.47 to 1.60 (AAPC 4.97, 95% CI: 4.31 to 5.63%), demonstrating epidemiological characteristics distinct from other countries.

During 2010–2021, trends further diverged across countries. In the United States, ASIR remained relatively stable overall, increasing slightly from 17.50 to 17.76 (AAPC 0.40, 95% CI: −0.20 to 1.02%). Canada showed a significant upward trend, with ASIR rising to 10.31 (AAPC 3.20, 95% CI: 1.78 to 4.64%), whereas France remained largely stable (AAPC −0.05, 95% CI: −0.12 to 0.02%). Japan continued to exhibit a mild increase (AAPC 1.14, 95% CI: 0.77 to 1.52%).

Sex-stratified analysis showed that ASIR was consistently higher in males than females. For example, in France, male ASIR increased to 22.48 before 2010 and then showed a slight decline thereafter. Subtype analysis revealed that incidence rates for HIV/AIDS with drug-susceptible tuberculosis declined substantially across countries, such as in Germany from 1.48 to 0.25 (AAPC −10.47, 95% CI: −11.54 to −9.40%). In contrast, incidence rates for extensively drug-resistant tuberculosis remained close to zero or unavailable, indicating an extremely low burden in this age group (Table 1).

**Table 1:** Results of ASIR in G7 countries.

Cause	Country	Sex	Value_ (1990-2010)	PC	AAPC_UI_ (1990-2010)	Value_ (2010-2021)	PC	AAPC_UI_ (2010-2021)
HIV/AIDS	Canada	Both	9.7 (5.22, 13.72) → 6.8 (3.89, 10.39)	-29.9%	-1.01% (-1.63%, -0.39%)	6.8 (3.89, 10.39) → 10.31 (5.04, 15.77)	51.6%	3.2% (1.78%, 4.64%)
		Female	3.26 (1.48, 5.12) → 4.51 (2.12, 7.78)	38.3%	1.48% (0.4%, 2.57%)	4.51 (2.12, 7.78) → 5.75 (2.74, 8.93)	27.5%	1.89% (1.19%, 2.6%)
		Male	16.15 (8.86, 23.08) → 9.18 (5.47, 12.96)	-43.2%	-1.87% (-2.57%, -1.15%)	9.18 (5.47, 12.96) → 14.95 (6.83, 23.4)	62.9%	3.73% (1.91%, 5.57%)
	France	Both	9.91 (8.28, 12.44) → 14.38 (13.31, 15.49)	45.1%	3.03% (1.78%, 4.29%)	14.38 (13.31, 15.49) → 14.27 (13.06, 15.5)	-0.8%	-0.05% (-0.12%, 0.02%)
		Female	4.33 (3.39, 5.61) → 6.48 (5.41, 7.64)	49.7%	3.13% (1.88%, 4.4%)	6.48 (5.41, 7.64) → 6.42 (5.37, 7.56)	-0.9%	-0.06% (-0.12%, 0.01%)
		Male	15.59 (12.82, 19.97) → 22.48 (20.34, 24.59)	44.2%	3.01% (1.77%, 4.28%)	22.48 (20.34, 24.59) → 22.35 (19.9, 24.81)	-0.6%	-0.02% (-0.09%, 0.05%)
	Germany	Both	6.51 (5.24, 7.83) → 4.72 (3.12, 6.62)	-27.5%	-0.17% (-2.02%, 1.71%)	4.72 (3.12, 6.62) → 3.26 (2.12, 4.76)	-30.9%	-2.03% (-3.14%, -0.91%)
		Female	2.94 (2.1, 4.09) → 3.43 (1.74, 5.59)	16.7%	1.95% (0.46%, 3.47%)	3.43 (1.74, 5.59) → 2.03 (1.06, 3.44)	-40.8%	-3.64% (-4.63%, -2.63%)
		Male	9.95 (7.94, 12.38) → 5.97 (4.19, 8.73)	-40%	-1.04% (-2.99%, 0.96%)	5.97 (4.19, 8.73) → 4.44 (2.91, 6.84)	-25.6%	-1.25% (-2.45%, -0.03%)
	Italy	Both	13.98 (9.6, 21.73) → 8.08 (5.9, 10.76)	-42.2%	-1.31% (-2.3%, -0.3%)	8.08 (5.9, 10.76) → 5.05 (3.57, 6.73)	-37.5%	-3.75% (-4.45%, -3.04%)
		Female	6.94 (4.55, 9.8) → 5.18 (3.45, 7.58)	-25.4%	-0.31% (-1.15%, 0.53%)	5.18 (3.45, 7.58) → 3.18 (2.08, 4.46)	-38.6%	-3.84% (-4.74%, -2.93%)
		Male	21.07 (13.82, 34.4) → 11.04 (8.29, 14.72)	-47.6%	-1.69% (-2.76%, -0.62%)	11.04 (8.29, 14.72) → 6.91 (5.12, 9.37)	-37.4%	-3.78% (-4.43%, -3.13%)
	Japan	Both	0.47 (0.25, 0.76) → 1.6 (1.04, 2.24)	240.4%	4.97% (4.31%, 5.63%)	1.6 (1.04, 2.24) → 1.85 (0.81, 2.7)	15.6%	1.14% (0.77%, 1.52%)
		Female	0.25 (0.13, 0.42) → 0.97 (0.62, 1.32)	288%	5.73% (5.04%, 6.43%)	0.97 (0.62, 1.32) → 1.11 (0.51, 1.61)	14.4%	0.78% (0.38%, 1.19%)
		Male	0.69 (0.37, 1.1) → 2.22 (1.46, 3.05)	221.7%	4.67% (4.01%, 5.33%)	2.22 (1.46, 3.05) → 2.6 (1.11, 3.82)	17.1%	1.3% (0.86%, 1.74%)
	United Kingdom	Both	3.93 (3.08, 5.06) → 6.36 (4.29, 8.56)	61.8%	4.07% (1.92%, 6.28%)	6.36 (4.29, 8.56) → 6.55 (4.45, 8.84)	3%	0.49% (-0.13%, 1.11%)
		Female	1.66 (1.21, 2.24) → 3.89 (2.67, 5.23)	134.3%	4.96% (1.43%, 8.62%)	3.89 (2.67, 5.23) → 4.25 (2.82, 5.61)	9.3%	0.89% (-0.07%, 1.86%)
		Male	6.22 (4.9, 8.01) → 8.87 (5.92, 11.94)	42.6%	3.81% (2.2%, 5.44%)	8.87 (5.92, 11.94) → 8.96 (6.17, 12.2)	1%	0.39% (-0.21%, 1%)
	United States of America	Both	26.44 (18.3, 34.25) → 17.5 (10.66, 24.76)	-33.8%	-1.43% (-2.46%, -0.39%)	17.5 (10.66, 24.76) → 17.76 (9.2, 26.74)	1.5%	0.4% (-0.2%, 1.02%)
		Female	15.27 (10.86, 19.64) → 10.78 (6.63, 15.62)	-29.4%	-1.51% (-2.29%, -0.72%)	10.78 (6.63, 15.62) → 11.13 (5.71, 17.81)	3.2%	0.77% (0.12%, 1.43%)
		Male	37.67 (24.74, 49.85) → 24.22 (14.54, 35.08)	-35.7%	-1.4% (-2.56%, -0.22%)	24.22 (14.54, 35.08) → 24.31 (12.6, 37.62)	0.4%	0.21% (-0.4%, 0.82%)
HIV/AIDS - Drug-susceptible tuberculosis	Canada	Both	1.29 (1.06, 1.53) → 0.47 (0.41, 0.55)	-63.6%	-6.06% (-6.79%, -5.33%)	0.47 (0.41, 0.55) → 0.35 (0.3, 0.4)	-25.5%	-2.75% (-3.72%, -1.77%)
		Female	0.24 (0.2, 0.28) → 0.25 (0.21, 0.29)	4.2%	-1.29% (-2.27%, -0.31%)	0.25 (0.21, 0.29) → 0.2 (0.17, 0.22)	-20%	-2.18% (-3.02%, -1.34%)
		Male	2.35 (1.91, 2.79) → 0.71 (0.61, 0.83)	-69.8%	-6.98% (-7.72%, -6.24%)	0.71 (0.61, 0.83) → 0.51 (0.44, 0.59)	-28.2%	-2.97% (-3.98%, -1.94%)
	France	Both	3.74 (3.18, 4.4) → 0.62 (0.53, 0.72)	-83.4%	-10.08% (-10.99%, -9.16%)	0.62 (0.53, 0.72) → 0.36 (0.31, 0.41)	-41.9%	-4.78% (-5.12%, -4.44%)
		Female	1.4 (1.19, 1.64) → 0.3 (0.25, 0.34)	-78.6%	-8.92% (-9.81%, -8.02%)	0.3 (0.25, 0.34) → 0.18 (0.15, 0.21)	-40%	-4.32% (-4.61%, -4.03%)
		Male	6.15 (5.23, 7.24) → 0.96 (0.82, 1.11)	-84.4%	-10.36% (-11.28%, -9.43%)	0.96 (0.82, 1.11) → 0.54 (0.47, 0.62)	-43.7%	-4.9% (-5.27%, -4.54%)
	Germany	Both	1.48 (1.25, 1.74) → 0.25 (0.22, 0.29)	-83.1%	-10.47% (-11.54%, -9.4%)	0.25 (0.22, 0.29) → 0.18 (0.15, 0.21)	-28%	-2.8% (-3.13%, -2.46%)
		Female	0.52 (0.44, 0.62) → 0.14 (0.12, 0.16)	-73.1%	-8.46% (-9.56%, -7.35%)	0.14 (0.12, 0.16) → 0.11 (0.09, 0.13)	-21.4%	-2.05% (-2.59%, -1.52%)
		Male	2.4 (2.04, 2.83) → 0.36 (0.31, 0.42)	-85%	-10.97% (-12.02%, -9.91%)	0.36 (0.31, 0.42) → 0.25 (0.21, 0.29)	-30.6%	-3.07% (-3.33%, -2.82%)

	Italy	Both	0.83 (0.7, 0.98) → 0.23 (0.19, 0.27)	-72.3%	-8.39% (-9.62%, -7.15%)	0.23 (0.19, 0.27) → 0.12 (0.1, 0.15)	-47.8%	-5.75% (-6.04%, -5.46%)
		Female	0.35 (0.29, 0.41) → 0.13 (0.11, 0.16)	-62.9%	-6.79% (-8.01%, -5.56%)	0.13 (0.11, 0.16) → 0.08 (0.07, 0.09)	-38.5%	-5.06% (-5.36%, -4.75%)
		Male	1.32 (1.11, 1.55) → 0.32 (0.27, 0.38)	-75.8%	-8.89% (-10.13%, -7.64%)	0.32 (0.27, 0.38) → 0.17 (0.14, 0.2)	-46.9%	-6.06% (-6.35%, -5.77%)
	Japan	Both	0.17 (0.14, 0.2) → 0.13 (0.11, 0.15)	-23.5%	-2.14% (-3.24%, -1.02%)	0.13 (0.11, 0.15) → 0.06 (0.05, 0.07)	-53.8%	-6.32% (-6.88%, -5.76%)
		Female	0.09 (0.07, 0.1) → 0.07 (0.06, 0.08)	-22.2%	-1.68% (-2.79%, -0.57%)	0.07 (0.06, 0.08) → 0.04 (0.03, 0.04)	-42.9%	-5.39% (-5.83%, -4.94%)
		Male	0.26 (0.22, 0.3) → 0.18 (0.15, 0.21)	-30.8%	-2.29% (-3.39%, -1.18%)	0.18 (0.15, 0.21) → 0.08 (0.07, 0.1)	-55.6%	-6.7% (-7.31%, -6.08%)
	United Kingdom	Both	1.02 (0.85, 1.2) → 0.62 (0.53, 0.73)	-39.2%	-3.11% (-4.22%, -1.99%)	0.62 (0.53, 0.73) → 0.25 (0.21, 0.29)	-59.7%	-9.17% (-9.97%, -8.37%)
		Female	0.29 (0.24, 0.34) → 0.44 (0.37, 0.51)	51.7%	1.65% (0.92%, 2.38%)	0.44 (0.37, 0.51) → 0.17 (0.15, 0.2)	-61.4%	-9.11% (-9.9%, -8.32%)
		Male	1.75 (1.46, 2.06) → 0.82 (0.7, 0.96)	-53.1%	-4.69% (-5.99%, -3.38%)	0.82 (0.7, 0.96) → 0.32 (0.27, 0.38)	-61%	-9.16% (-9.96%, -8.34%)
	United States of America	Both	1.54 (1.29, 1.83) → 0.28 (0.24, 0.32)	-81.8%	-9.32% (-10.19%, -8.44%)	0.28 (0.24, 0.32) → 0.12 (0.1, 0.14)	-57.1%	-8.34% (-9.05%, -7.63%)
		Female	0.53 (0.44, 0.62) → 0.14 (0.12, 0.16)	-73.6%	-7.4% (-8.27%, -6.52%)	0.14 (0.12, 0.16) → 0.06 (0.05, 0.07)	-57.1%	-7.73% (-8.45%, -7%)
Male		2.57 (2.15, 3.06) → 0.42 (0.36, 0.48)	-83.7%	-9.81% (-10.69%, -8.92%)	0.42 (0.36, 0.48) → 0.17 (0.14, 0.2)	-59.5%	-8.59% (-9.3%, -7.88%)
HIV/AIDS - Extensively drug-resistant tuberculosis	Canada	Both	0 (0, 0) → 0 (0, 0)	NaN%	31.28% (4.77%, 64.49%)	0 (0, 0) → 0 (0, 0)	NaN%	2.43% (0.25%, 4.65%)
		Female	0 (0, 0) → 0 (0, 0)	NaN%	34.91% (10.29%, 65.02%)	0 (0, 0) → 0 (0, 0)	NaN%	2.92% (1.2%, 4.68%)
		Male	0 (0, 0) → 0 (0, 0)	NaN%	31.13% (3.68%, 65.85%)	0 (0, 0) → 0 (0, 0)	NaN%	2.25% (-0.1%, 4.65%)
	France	Both	0 (0, 0) → 0 (0, 0)	NaN%	40.81% (10.7%, 79.1%)	0 (0, 0) → 0 (0, 0)	NaN%	-2.94% (-3.53%, -2.34%)
		Female	0 (0, 0) → 0 (0, 0)	NaN%	42.13% (13.42%, 78.1%)	0 (0, 0) → 0 (0, 0)	NaN%	-2.43% (-3.18%, -1.67%)
		Male	0 (0, 0) → 0 (0, 0)	NaN%	40.83% (9.88%, 80.5%)	0 (0, 0) → 0 (0, 0)	NaN%	-3.11% (-3.68%, -2.55%)
	Germany	Both	0 (0, 0) → 0 (0, 0)	NaN%	35.92% (8.78%, 69.82%)	0 (0, 0) → 0 (0, 0)	NaN%	3.6% (1.36%, 5.89%)
		Female	0 (0, 0) → 0 (0, 0)	NaN%	36.85% (11.17%, 68.46%)	0 (0, 0) → 0 (0, 0)	NaN%	5.09% (2.78%, 7.45%)
		Male	0 (0, 0) → 0 (0, 0)	NaN%	36.11% (8.15%, 71.3%)	0 (0, 0) → 0 (0, 0)	NaN%	3.16% (0.93%, 5.43%)
	Italy	Both	0 (0, 0) → 0 (0, 0)	NaN%	38.63% (10.42%, 74.06%)	0 (0, 0) → 0 (0, 0)	NaN%	-4.05% (-5.64%, -2.43%)
		Female	0 (0, 0) → 0 (0, 0)	NaN%	38.03% (11.29%, 71.21%)	0 (0, 0) → 0 (0, 0)	NaN%	-3.31% (-4.69%, -1.92%)
		Male	0 (0, 0) → 0 (0, 0)	NaN%	39.26% (10.17%, 76.02%)	0 (0, 0) → 0 (0, 0)	NaN%	-4.32% (-5.99%, -2.63%)
	Japan	Both	0 (0, 0) → 0 (0, 0)	NaN%	34.27% (9.87%, 64.09%)	0 (0, 0) → 0 (0, 0)	NaN%	4.33% (3.66%, 5.01%)
		Female	0 (0, 0) → 0 (0, 0)	NaN%	32.48% (9.48%, 60.32%)	0 (0, 0) → 0 (0, 0)	NaN%	6.94% (6.07%, 7.83%)
		Male	0 (0, 0) → 0 (0, 0)	NaN%	35.2% (9.96%, 66.23%)	0 (0, 0) → 0 (0, 0)	NaN%	3.3% (2.69%, 3.93%)
	United Kingdom	Both	0 (0, 0) → 0 (0, 0)	NaN%	33.2% (7.48%, 65.07%)	0 (0, 0) → 0 (0, 0)	NaN%	-2.17% (-3.81%, -0.5%)
		Female	0 (0, 0) → 0 (0, 0)	NaN%	38.28% (13.67%, 68.21%)	0 (0, 0) → 0 (0, 0)	NaN%	-1.5% (-3.06%, 0.08%)
		Male	0 (0, 0) → 0 (0, 0)	NaN%	31.73% (5.36%, 64.72%)	0 (0, 0) → 0 (0, 0)	NaN%	-2.49% (-4.31%, -0.63%)

	United States of America	Both	0 (0, 0) → 0 (0, 0)	NaN%	23.71% (-2.06%, 56.24%)	0 (0, 0) → 0 (0, 0)	NaN%	2.92% (0.88%, 5%)
		Female	0 (0, 0) → 0 (0, 0)	NaN%	25.8% (1.3%, 56.23%)	0 (0, 0) → 0 (0, 0)	NaN%	2.52% (0.47%, 4.62%)
Male		0 (0, 0) → 0 (0, 0)	NaN%	23.44% (-3.06%, 57.19%)	0 (0, 0) → 0 (0, 0)	NaN%	3.04% (1.01%, 5.11%)
HIV/AIDS - Multidrug-resistant tuberculosis without extensive drug resistance	Canada	Both	0.01 (0, 0.04) → 0 (0, 0.01)	-100%	-7.99% (-9.18%, -6.79%)	0 (0, 0.01) → 0 (0, 0.01)	NaN%	-2.29% (-3.51%, -1.05%)
		Female	0 (0, 0.01) → 0 (0, 0)	NaN%	-2.41% (-3.76%, -1.03%)	0 (0, 0) → 0 (0, 0)	NaN%	-1.95% (-2.85%, -1.05%)
		Male	0.02 (0.01, 0.06) → 0.01 (0, 0.01)	-50%	-9.04% (-10.28%, -7.79%)	0.01 (0, 0.01) → 0 (0, 0.01)	-100%	-2.42% (-3.77%, -1.05%)
	France	Both	0.03 (0.01, 0.1) → 0.02 (0.01, 0.02)	-33.3%	-5.04% (-6.71%, -3.35%)	0.02 (0.01, 0.02) → 0.01 (0, 0.01)	-50%	-7.1% (-7.71%, -6.49%)
		Female	0.01 (0, 0.03) → 0.01 (0.01, 0.01)	0%	-2.24% (-4.23%, -0.2%)	0.01 (0.01, 0.01) → 0 (0, 0.01)	-100%	-6.79% (-7.54%, -6.04%)
		Male	0.05 (0.01, 0.17) → 0.02 (0.02, 0.03)	-60%	-5.75% (-7.37%, -4.1%)	0.02 (0.02, 0.03) → 0.01 (0.01, 0.02)	-50%	-7.19% (-7.78%, -6.6%)
	Germany	Both	0.01 (0, 0.04) → 0.01 (0, 0.01)	0%	-6.42% (-7.66%, -5.16%)	0.01 (0, 0.01) → 0.01 (0, 0.01)	0%	0.48% (-1.03%, 2.02%)
		Female	0 (0, 0.01) → 0 (0, 0)	NaN%	-3.92% (-5.22%, -2.6%)	0 (0, 0) → 0 (0, 0.01)	NaN%	1.97% (0.34%, 3.64%)
		Male	0.02 (0, 0.07) → 0.01 (0.01, 0.01)	-50%	-7.01% (-8.29%, -5.71%)	0.01 (0.01, 0.01) → 0.01 (0, 0.02)	0%	0% (-1.5%, 1.53%)
	Italy	Both	0.01 (0, 0.02) → 0.01 (0.01, 0.01)	0%	-1.06% (-3.88%, 1.84%)	0.01 (0.01, 0.01) → 0 (0, 0)	-100%	-9.05% (-9.88%, -8.21%)
		Female	0 (0, 0.01) → 0 (0, 0)	NaN%	0.91% (-2.2%, 4.11%)	0 (0, 0) → 0 (0, 0)	NaN%	-8.43% (-9.03%, -7.82%)
		Male	0.01 (0, 0.04) → 0.01 (0.01, 0.01)	0%	-1.63% (-4.38%, 1.2%)	0.01 (0.01, 0.01) → 0 (0, 0.01)	-100%	-9.32% (-10.25%, -8.39%)
	Japan	Both	0 (0, 0) → 0 (0, 0)	NaN%	-2.32% (-6.19%, 1.7%)	0 (0, 0) → 0 (0, 0)	NaN%	-0.59% (-1.17%, -0.01%)
		Female	0 (0, 0) → 0 (0, 0)	NaN%	-2.63% (-6.51%, 1.4%)	0 (0, 0) → 0 (0, 0)	NaN%	1.54% (0.8%, 2.28%)
		Male	0 (0, 0) → 0 (0, 0)	NaN%	-2.2% (-6.06%, 1.82%)	0 (0, 0) → 0 (0, 0)	NaN%	-1.46% (-1.98%, -0.93%)
	United Kingdom	Both	0.01 (0, 0.02) → 0.01 (0, 0.01)	0%	-4.75% (-6.66%, -2.81%)	0.01 (0, 0.01) → 0 (0, 0)	-100%	-9% (-9.76%, -8.24%)
		Female	0 (0, 0.01) → 0 (0, 0.01)	NaN%	1.15% (-0.35%, 2.67%)	0 (0, 0.01) → 0 (0, 0)	NaN%	-8.94% (-9.68%, -8.2%)
		Male	0.01 (0, 0.04) → 0.01 (0.01, 0.01)	0%	-6.64% (-8.73%, -4.51%)	0.01 (0.01, 0.01) → 0 (0, 0.01)	-100%	-8.99% (-9.76%, -8.21%)
	United States of America	Both	0.05 (0.02, 0.08) → 0 (0, 0)	-100%	-15.26% (-16.91%, -13.58%)	0 (0, 0) → 0 (0, 0)	NaN%	-3.91% (-5.88%, -1.9%)
		Female	0.01 (0.01, 0.02) → 0 (0, 0)	-100%	-12.24% (-13.42%, -11.06%)	0 (0, 0) → 0 (0, 0)	NaN%	-3.7% (-5.59%, -1.78%)
		Male	0.08 (0.04, 0.13) → 0 (0, 0)	-100%	-16.03% (-17.8%, -14.23%)	0 (0, 0) → 0 (0, 0.01)	NaN%	-4.01% (-6.01%, -1.97%)
HIV/AIDS resulting in other diseases	Canada	Both	9.7 (5.22, 13.72) → 6.8 (3.89, 10.39)	-29.9%	-1.01% (-1.63%, -0.39%)	6.8 (3.89, 10.39) → 10.31 (5.04, 15.77)	51.6%	3.2% (1.78%, 4.64%)
		Female	3.26 (1.48, 5.12) → 4.51 (2.12, 7.78)	38.3%	1.48% (0.4%, 2.57%)	4.51 (2.12, 7.78) → 5.75 (2.74, 8.93)	27.5%	1.89% (1.19%, 2.6%)
		Male	16.15 (8.86, 23.08) → 9.18 (5.47, 12.96)	-43.2%	-1.87% (-2.57%, -1.15%)	9.18 (5.47, 12.96) → 14.95 (6.83, 23.4)	62.9%	3.73% (1.91%, 5.57%)

	France	Both	9.91 (8.28, 12.44) → 14.38 (13.31, 15.49)	45.1%	3.03% (1.78%, 4.29%)	14.38 (13.31, 15.49) → 14.27 (13.06, 15.5)	-0.8%	-0.05% (-0.12%, 0.02%)
		Female	4.33 (3.39, 5.61) → 6.48 (5.41, 7.64)	49.7%	3.13% (1.88%, 4.4%)	6.48 (5.41, 7.64) → 6.42 (5.37, 7.56)	-0.9%	-0.06% (-0.12%, 0.01%)
		Male	15.59 (12.82, 19.97) → 22.48 (20.34, 24.59)	44.2%	3.01% (1.77%, 4.28%)	22.48 (20.34, 24.59) → 22.35 (19.9, 24.81)	-0.6%	-0.02% (-0.09%, 0.05%)
	Germany	Both	6.51 (5.24, 7.83) → 4.72 (3.12, 6.62)	-27.5%	-0.17% (-2.02%, 1.71%)	4.72 (3.12, 6.62) → 3.26 (2.12, 4.76)	-30.9%	-2.03% (-3.14%, -0.91%)
		Female	2.94 (2.1, 4.09) → 3.43 (1.74, 5.59)	16.7%	1.95% (0.46%, 3.47%)	3.43 (1.74, 5.59) → 2.03 (1.06, 3.44)	-40.8%	-3.64% (-4.63%, -2.63%)
		Male	9.95 (7.94, 12.38) → 5.97 (4.19, 8.73)	-40%	-1.04% (-2.99%, 0.96%)	5.97 (4.19, 8.73) → 4.44 (2.91, 6.84)	-25.6%	-1.25% (-2.45%, -0.03%)
	Italy	Both	13.98 (9.6, 21.73) → 8.08 (5.9, 10.76)	-42.2%	-1.31% (-2.3%, -0.3%)	8.08 (5.9, 10.76) → 5.05 (3.57, 6.73)	-37.5%	-3.75% (-4.45%, -3.04%)
		Female	6.94 (4.55, 9.8) → 5.18 (3.45, 7.58)	-25.4%	-0.31% (-1.15%, 0.53%)	5.18 (3.45, 7.58) → 3.18 (2.08, 4.46)	-38.6%	-3.84% (-4.74%, -2.93%)
		Male	21.07 (13.82, 34.4) → 11.04 (8.29, 14.72)	-47.6%	-1.69% (-2.76%, -0.62%)	11.04 (8.29, 14.72) → 6.91 (5.12, 9.37)	-37.4%	-3.78% (-4.43%, -3.13%)
	Japan	Both	0.47 (0.25, 0.76) → 1.6 (1.04, 2.24)	240.4%	4.97% (4.31%, 5.63%)	1.6 (1.04, 2.24) → 1.85 (0.81, 2.7)	15.6%	1.14% (0.77%, 1.52%)
		Female	0.25 (0.13, 0.42) → 0.97 (0.62, 1.32)	288%	5.73% (5.04%, 6.43%)	0.97 (0.62, 1.32) → 1.11 (0.51, 1.61)	14.4%	0.78% (0.38%, 1.19%)
		Male	0.69 (0.37, 1.1) → 2.22 (1.46, 3.05)	221.7%	4.67% (4.01%, 5.33%)	2.22 (1.46, 3.05) → 2.6 (1.11, 3.82)	17.1%	1.3% (0.86%, 1.74%)
	United Kingdom	Both	3.93 (3.08, 5.06) → 6.36 (4.29, 8.56)	61.8%	4.07% (1.92%, 6.28%)	6.36 (4.29, 8.56) → 6.55 (4.45, 8.84)	3%	0.49% (-0.13%, 1.11%)
		Female	1.66 (1.21, 2.24) → 3.89 (2.67, 5.23)	134.3%	4.96% (1.43%, 8.62%)	3.89 (2.67, 5.23) → 4.25 (2.82, 5.61)	9.3%	0.89% (-0.07%, 1.86%)
		Male	6.22 (4.9, 8.01) → 8.87 (5.92, 11.94)	42.6%	3.81% (2.2%, 5.44%)	8.87 (5.92, 11.94) → 8.96 (6.17, 12.2)	1%	0.39% (-0.21%, 1%)
	United States of America	Both	26.44 (18.3, 34.25) → 17.5 (10.66, 24.76)	-33.8%	-1.43% (-2.46%, -0.39%)	17.5 (10.66, 24.76) → 17.76 (9.2, 26.74)	1.5%	0.4% (-0.2%, 1.02%)
		Female	15.27 (10.86, 19.64) → 10.78 (6.63, 15.62)	-29.4%	-1.51% (-2.29%, -0.72%)	10.78 (6.63, 15.62) → 11.13 (5.71, 17.81)	3.2%	0.77% (0.12%, 1.43%)
Male		37.67 (24.74, 49.85) → 24.22 (14.54, 35.08)	-35.7%	-1.4% (-2.56%, -0.22%)	24.22 (14.54, 35.08) → 24.31 (12.6, 37.62)	0.4%	0.21% (-0.4%, 0.82%)

### 3.3 Age-standardized mortality rate: sustained decline driven by treatment advances

Following the trends in incidence, ASMR further reflected improvements in disease outcomes. From 1990 to 2010, HIV/AIDS mortality declined significantly in most G7 countries; for example, Canada decreased from 2.61 to 0.76 (AAPC −7.90, 95% CI: −9.34 to −6.43%), consistent with the widespread implementation of ART. In contrast, Japan showed an increasing trend, with ASMR rising from 0.04 to 0.12 (AAPC 4.94, 95% CI: 3.71 to 6.19%).

During 2010–2021, ASMR continued to decline across countries, although at a slower pace. In the United States, ASMR decreased from 9.62 to 1.46 (AAPC −4.92, 95% CI: −5.45 to −4.38%). Similar declining trends were observed in Canada (AAPC −6.91, 95% CI: −8.16 to −5.65%), France (AAPC −6.58, 95% CI: −6.96 to −6.19%), and Italy (AAPC −6.53, 95% CI: −7.00 to −6.06%), whereas Japan showed a slight decline after 2010 (AAPC −0.70, 95% CI: −1.03 to −0.37%).

Sex-stratified analysis showed that ASMR was consistently higher in males than females across all G7 countries. For example, in Italy, male ASMR decreased from 4.85 to 0.91, whereas female ASMR declined from 1.04 to 0.30. Similarly, in the United States, male ASMR decreased from 17.07 to 2.13, compared with a decline from 2.36 to 0.81 in females. This male predominance in mortality was observed consistently across countries, although the magnitude of the sex gap varied. At the subtype level, ASMR for HIV/AIDS with drug-susceptible tuberculosis declined substantially across countries (e.g., Canada from 0.42 to 0.07, AAPC −9.85, 95% CI: −10.73 to −8.97%), whereas ASMR for multidrug-resistant and extensively drug-resistant tuberculosis remained extremely low or negligible (Table 2).

**Table 2:** Results of ASMR in G7 countries.

Cause	Country	Sex	Value_ (1990-2010)	PC	AAPC_UI_ (1990-2010)	Value_ (2010-2021)	PC	AAPC_UI_ (2010-2021)
HIV/AIDS	Canada	Both	2.61 (2.31, 2.92) → 0.76 (0.69, 0.82)	-70.9%	-7.9% (-9.34%, -6.43%)	0.76 (0.69, 0.82) → 0.36 (0.32, 0.4)	-52.6%	-6.91% (-8.16%, -5.65%)
		Female	0.4 (0.35, 0.44) → 0.35 (0.31, 0.39)	-12.5%	-2.07% (-3.35%, -0.76%)	0.35 (0.31, 0.39) → 0.18 (0.16, 0.21)	-48.6%	-6.26% (-7.01%, -5.5%)
		Male	4.86 (4.24, 5.45) → 1.18 (1.05, 1.31)	-75.7%	-8.79% (-10.32%, -7.24%)	1.18 (1.05, 1.31) → 0.55 (0.48, 0.61)	-53.4%	-7.13% (-8.55%, -5.7%)
	France	Both	4.55 (4.16, 4.97) → 0.88 (0.81, 0.96)	-80.7%	-10.44% (-12.15%, -8.7%)	0.88 (0.81, 0.96) → 0.43 (0.37, 0.49)	-51.1%	-6.58% (-6.96%, -6.19%)
		Female	1.29 (1.16, 1.44) → 0.51 (0.45, 0.57)	-60.5%	-7.64% (-9.47%, -5.77%)	0.51 (0.45, 0.57) → 0.26 (0.22, 0.31)	-49%	-6.03% (-6.23%, -5.83%)
		Male	7.83 (7.03, 8.64) → 1.28 (1.12, 1.43)	-83.7%	-11.12% (-12.81%, -9.39%)	1.28 (1.12, 1.43) → 0.61 (0.5, 0.74)	-52.3%	-6.77% (-7.27%, -6.26%)
	Germany	Both	1.75 (1.58, 1.91) → 0.55 (0.51, 0.6)	-68.6%	-8.38% (-10.07%, -6.66%)	0.55 (0.51, 0.6) → 0.31 (0.28, 0.33)	-43.6%	-4.48% (-5.85%, -3.09%)
		Female	0.52 (0.47, 0.58) → 0.27 (0.24, 0.3)	-48.1%	-5.88% (-7.34%, -4.4%)	0.27 (0.24, 0.3) → 0.17 (0.14, 0.19)	-37%	-3.09% (-4.57%, -1.58%)
		Male	2.97 (2.64, 3.29) → 0.84 (0.75, 0.92)	-71.7%	-8.95% (-10.73%, -7.14%)	0.84 (0.75, 0.92) → 0.45 (0.39, 0.51)	-46.4%	-4.89% (-6.24%, -3.53%)
	Italy	Both	2.94 (2.76, 3.12) → 1.19 (1.13, 1.25)	-59.5%	-7.92% (-9.95%, -5.85%)	1.19 (1.13, 1.25) → 0.6 (0.57, 0.63)	-49.6%	-6.53% (-7%, -6.06%)
		Female	1.04 (0.96, 1.12) → 0.57 (0.53, 0.61)	-45.2%	-6.8% (-8.92%, -4.63%)	0.57 (0.53, 0.61) → 0.3 (0.28, 0.33)	-47.4%	-5.77% (-6.09%, -5.44%)
		Male	4.85 (4.49, 5.2) → 1.85 (1.73, 1.95)	-61.9%	-8.16% (-10.19%, -6.09%)	1.85 (1.73, 1.95) → 0.91 (0.84, 0.97)	-50.8%	-6.81% (-7.41%, -6.2%)
	Japan	Both	0.04 (0.04, 0.05) → 0.12 (0.12, 0.13)	200%	4.94% (3.71%, 6.19%)	0.12 (0.12, 0.13) → 0.11 (0.11, 0.12)	-8.3%	-0.7% (-1.03%, -0.37%)
		Female	0.03 (0.02, 0.03) → 0.06 (0.06, 0.07)	100%	4.19% (3.25%, 5.14%)	0.06 (0.06, 0.07) → 0.07 (0.07, 0.08)	16.7%	1.74% (1.2%, 2.29%)
		Male	0.06 (0.06, 0.07) → 0.19 (0.18, 0.2)	216.7%	5.17% (3.84%, 6.52%)	0.19 (0.18, 0.2) → 0.15 (0.14, 0.16)	-21.1%	-1.69% (-1.98%, -1.4%)
	United Kingdom	Both	0.82 (0.79, 0.86) → 0.46 (0.44, 0.47)	-43.9%	-5.17% (-6.97%, -3.34%)	0.46 (0.44, 0.47) → 0.3 (0.29, 0.31)	-34.8%	-3.31% (-4.03%, -2.59%)
		Female	0.15 (0.14, 0.15) → 0.32 (0.3, 0.33)	113.3%	2.18% (0.51%, 3.88%)	0.32 (0.3, 0.33) → 0.22 (0.21, 0.23)	-31.2%	-2.59% (-3.92%, -1.24%)
		Male	1.51 (1.45, 1.58) → 0.6 (0.57, 0.62)	-60.3%	-7.14% (-9.07%, -5.17%)	0.6 (0.57, 0.62) → 0.38 (0.37, 0.4)	-36.7%	-3.65% (-4.29%, -3.01%)
	United States of America	Both	9.62 (9.3, 9.96) → 2.54 (2.46, 2.61)	-73.6%	-8.09% (-9.27%, -6.88%)	2.54 (2.46, 2.61) → 1.46 (1.39, 1.51)	-42.5%	-4.92% (-5.45%, -4.38%)
		Female	2.36 (2.27, 2.44) → 1.49 (1.43, 1.55)	-36.9%	-3.74% (-5.06%, -2.41%)	1.49 (1.43, 1.55) → 0.81 (0.77, 0.85)	-45.6%	-5.37% (-5.87%, -4.87%)
		Male	17.07 (16.45, 17.76) → 3.62 (3.47, 3.76)	-78.8%	-9.19% (-10.46%, -7.9%)	3.62 (3.47, 3.76) → 2.13 (2.01, 2.23)	-41.2%	-4.76% (-5.3%, -4.22%)
HIV/AIDS - Drug-susceptible tuberculosis	Canada	Both	0.42 (0.26, 0.63) → 0.07 (0.04, 0.1)	-83.3%	-9.85% (-10.73%, -8.97%)	0.07 (0.04, 0.1) → 0.06 (0.04, 0.09)	-14.3%	-1.94% (-3.97%, 0.14%)
		Female	0.06 (0.04, 0.1) → 0.03 (0.02, 0.05)	-50%	-4.14% (-5.17%, -3.09%)	0.03 (0.02, 0.05) → 0.03 (0.02, 0.05)	0%	-1.24% (-2.84%, 0.38%)
		Male	0.79 (0.48, 1.17) → 0.1 (0.06, 0.16)	-87.3%	-10.72% (-11.68%, -9.75%)	0.1 (0.06, 0.16) → 0.09 (0.05, 0.14)	-10%	-2.17% (-4.36%, 0.06%)
	France	Both	1.76 (1.21, 2.12) → 0.28 (0.17, 0.37)	-84.1%	-11.03% (-12.42%, -9.61%)	0.28 (0.17, 0.37) → 0.11 (0.06, 0.15)	-60.7%	-8.06% (-8.9%, -7.21%)
		Female	0.5 (0.35, 0.61) → 0.16 (0.1, 0.21)	-68%	-8.26% (-9.89%, -6.6%)	0.16 (0.1, 0.21) → 0.06 (0.04, 0.09)	-62.5%	-7.55% (-8.47%, -6.61%)
		Male	3.04 (2.08, 3.69) → 0.41 (0.25, 0.54)	-86.5%	-11.69% (-13.06%, -10.3%)	0.41 (0.25, 0.54) → 0.15 (0.09, 0.22)	-63.4%	-8.23% (-9.08%, -7.38%)
	Germany	Both	0.56 (0.35, 0.77) → 0.06 (0.04, 0.09)	-89.3%	-12.46% (-13.74%, -11.15%)	0.06 (0.04, 0.09) → 0.03 (0.02, 0.05)	-50%	-4.73% (-5.89%, -3.56%)
		Female	0.16 (0.11, 0.22) → 0.03 (0.02, 0.04)	-81.2%	-10.06% (-11.12%, -8.99%)	0.03 (0.02, 0.04) → 0.02 (0.01, 0.03)	-33.3%	-3.36% (-4.64%, -2.06%)
		Male	0.95 (0.6, 1.3) → 0.09 (0.06, 0.14)	-90.5%	-13% (-14.38%, -11.6%)	0.09 (0.06, 0.14) → 0.05 (0.03, 0.08)	-44.4%	-5.14% (-6.28%, -3.99%)

	Italy	Both	0.19 (0.12, 0.29) → 0.04 (0.02, 0.06)	-78.9%	-10.3% (-12.04%, -8.53%)	0.04 (0.02, 0.06) → 0.02 (0.01, 0.03)	-50%	-5.86% (-7.03%, -4.67%)
		Female	0.07 (0.04, 0.1) → 0.02 (0.01, 0.03)	-71.4%	-9.19% (-11%, -7.35%)	0.02 (0.01, 0.03) → 0.01 (0.01, 0.02)	-50%	-5.12% (-6.18%, -4.05%)
		Male	0.32 (0.19, 0.48) → 0.06 (0.04, 0.1)	-81.2%	-10.54% (-12.29%, -8.76%)	0.06 (0.04, 0.1) → 0.03 (0.02, 0.05)	-50%	-6.12% (-7.36%, -4.87%)
	Japan	Both	0.02 (0.02, 0.02) → 0.04 (0.03, 0.05)	100%	3.53% (2.2%, 4.88%)	0.04 (0.03, 0.05) → 0.03 (0.02, 0.04)	-25%	-1.28% (-2.2%, -0.35%)
		Female	0.01 (0.01, 0.01) → 0.02 (0.01, 0.03)	100%	2.68% (1.64%, 3.73%)	0.02 (0.01, 0.03) → 0.02 (0.01, 0.03)	0%	1.19% (0.08%, 2.31%)
		Male	0.03 (0.02, 0.03) → 0.06 (0.04, 0.08)	100%	3.79% (2.36%, 5.25%)	0.06 (0.04, 0.08) → 0.05 (0.03, 0.06)	-16.7%	-2.24% (-3.09%, -1.39%)
	United Kingdom	Both	0.21 (0.13, 0.29) → 0.08 (0.05, 0.12)	-61.9%	-5.88% (-6.78%, -4.97%)	0.08 (0.05, 0.12) → 0.04 (0.02, 0.06)	-50%	-5.71% (-7.08%, -4.32%)
		Female	0.04 (0.02, 0.05) → 0.05 (0.03, 0.08)	25%	1.23% (-0.15%, 2.64%)	0.05 (0.03, 0.08) → 0.03 (0.02, 0.04)	-40%	-5.01% (-6.55%, -3.45%)
		Male	0.38 (0.23, 0.54) → 0.1 (0.06, 0.16)	-73.7%	-7.81% (-8.84%, -6.77%)	0.1 (0.06, 0.16) → 0.05 (0.03, 0.08)	-50%	-6.04% (-7.49%, -4.56%)
	United States of America	Both	0.38 (0.24, 0.57) → 0.05 (0.03, 0.07)	-86.8%	-11.33% (-12.15%, -10.51%)	0.05 (0.03, 0.07) → 0.04 (0.02, 0.06)	-20%	-1.7% (-3.57%, 0.22%)
		Female	0.09 (0.06, 0.14) → 0.03 (0.02, 0.04)	-66.7%	-7.26% (-8.25%, -6.26%)	0.03 (0.02, 0.04) → 0.02 (0.01, 0.03)	-33.3%	-2.05% (-3.94%, -0.12%)
Male		0.68 (0.42, 1.01) → 0.07 (0.04, 0.1)	-89.7%	-12.34% (-13.24%, -11.44%)	0.07 (0.04, 0.1) → 0.06 (0.03, 0.09)	-14.3%	-1.59% (-3.46%, 0.31%)
HIV/AIDS - Extensively drug-resistant tuberculosis	Canada	Both	0 (0, 0) → 0 (0, 0)	NaN%	73.07% (25.13%, 139.36%)	0 (0, 0) → 0 (0, 0)	NaN%	2.49% (0.3%, 4.73%)
		Female	0 (0, 0) → 0 (0, 0)	NaN%	72.29% (28.96%, 130.18%)	0 (0, 0) → 0 (0, 0)	NaN%	3.08% (1.26%, 4.94%)
		Male	0 (0, 0) → 0 (0, 0)	NaN%	75.1% (24.94%, 145.41%)	0 (0, 0) → 0 (0, 0)	NaN%	2.28% (-0.06%, 4.68%)
	France	Both	0 (0, 0) → 0 (0, 0)	NaN%	86.64% (31.66%, 164.57%)	0 (0, 0) → 0 (0, 0)	NaN%	-2.94% (-3.53%, -2.34%)
		Female	0 (0, 0) → 0 (0, 0)	NaN%	84.52% (32.97%, 156.05%)	0 (0, 0) → 0 (0, 0)	NaN%	-2.43% (-3.18%, -1.67%)
		Male	0 (0, 0) → 0 (0, 0.01)	NaN%	88.69% (31.67%, 170.4%)	0 (0, 0.01) → 0 (0, 0)	NaN%	-3.11% (-3.68%, -2.55%)
	Germany	Both	0 (0, 0) → 0 (0, 0)	NaN%	78% (29.03%, 145.56%)	0 (0, 0) → 0 (0, 0)	NaN%	3.57% (1.32%, 5.88%)
		Female	0 (0, 0) → 0 (0, 0)	NaN%	75.43% (29.89%, 136.92%)	0 (0, 0) → 0 (0, 0)	NaN%	5.04% (2.72%, 7.42%)
		Male	0 (0, 0) → 0 (0, 0)	NaN%	80.11% (29.21%, 151.06%)	0 (0, 0) → 0 (0, 0)	NaN%	3.13% (0.89%, 5.42%)
	Italy	Both	0 (0, 0) → 0 (0, 0)	NaN%	82.13% (31.2%, 152.83%)	0 (0, 0) → 0 (0, 0)	NaN%	-4.06% (-5.72%, -2.37%)
		Female	0 (0, 0) → 0 (0, 0)	NaN%	77.96% (30.49%, 142.7%)	0 (0, 0) → 0 (0, 0)	NaN%	-3.34% (-4.97%, -1.69%)
		Male	0 (0, 0) → 0 (0, 0)	NaN%	84.81% (31.85%, 159.05%)	0 (0, 0) → 0 (0, 0)	NaN%	-4.32% (-6.01%, -2.61%)
	Japan	Both	0 (0, 0) → 0 (0, 0)	NaN%	71.97% (29.52%, 128.32%)	0 (0, 0) → 0 (0, 0)	NaN%	4.33% (3.65%, 5.01%)
		Female	0 (0, 0) → 0 (0, 0)	NaN%	67.17% (27.81%, 118.65%)	0 (0, 0) → 0 (0, 0)	NaN%	6.93% (6.05%, 7.82%)
		Male	0 (0, 0) → 0 (0, 0)	NaN%	74.67% (30.38%, 134.01%)	0 (0, 0) → 0 (0, 0)	NaN%	3.3% (2.68%, 3.93%)
	United Kingdom	Both	0 (0, 0) → 0 (0, 0)	NaN%	75.35% (29.05%, 138.26%)	0 (0, 0) → 0 (0, 0)	NaN%	-2.18% (-3.91%, -0.43%)
		Female	0 (0, 0) → 0 (0, 0)	NaN%	77.98% (34.49%, 135.54%)	0 (0, 0) → 0 (0, 0)	NaN%	-1.48% (-3.13%, 0.2%)
		Male	0 (0, 0) → 0 (0, 0)	NaN%	75.36% (27.45%, 141.27%)	0 (0, 0) → 0 (0, 0)	NaN%	-2.52% (-4.43%, -0.58%)

	United States of America	Both	0 (0, 0) → 0 (0, 0)	NaN%	65.88% (18.87%, 131.47%)	0 (0, 0) → 0 (0, 0)	NaN%	2.93% (0.88%, 5.01%)
		Female	0 (0, 0) → 0 (0, 0)	NaN%	65.32% (21.37%, 125.2%)	0 (0, 0) → 0 (0, 0)	NaN%	2.53% (0.48%, 4.62%)
Male		0 (0, 0) → 0 (0, 0)	NaN%	67.27% (18.49%, 136.13%)	0 (0, 0) → 0 (0, 0)	NaN%	3.05% (1.02%, 5.12%)
HIV/AIDS - Multidrug-resistant tuberculosis without extensive drug resistance	Canada	Both	0.01 (0, 0.03) → 0 (0, 0.01)	-100%	-8.94% (-10.22%, -7.65%)	0 (0, 0.01) → 0 (0, 0.01)	NaN%	-2.22% (-4.45%, 0.07%)
		Female	0 (0, 0) → 0 (0, 0)	NaN%	-3.5% (-5.01%, -1.96%)	0 (0, 0) → 0 (0, 0)	NaN%	-1.65% (-3.5%, 0.24%)
		Male	0.02 (0, 0.05) → 0 (0, 0.01)	-100%	-9.83% (-11.16%, -8.48%)	0 (0, 0.01) → 0 (0, 0.01)	NaN%	-2.42% (-4.8%, 0.02%)
	France	Both	0.02 (0, 0.08) → 0.01 (0, 0.03)	-50%	-5.03% (-6.7%, -3.33%)	0.01 (0, 0.03) → 0.01 (0, 0.01)	0%	-7.36% (-8.02%, -6.7%)
		Female	0.01 (0, 0.02) → 0.01 (0, 0.02)	0%	-2.2% (-4.21%, -0.14%)	0.01 (0, 0.02) → 0 (0, 0.01)	-100%	-6.88% (-7.63%, -6.12%)
		Male	0.04 (0.01, 0.14) → 0.02 (0.01, 0.04)	-50%	-5.74% (-7.37%, -4.09%)	0.02 (0.01, 0.04) → 0.01 (0, 0.02)	-50%	-7.53% (-8.19%, -6.87%)
	Germany	Both	0.01 (0, 0.04) → 0 (0, 0.01)	-100%	-6.45% (-7.69%, -5.19%)	0 (0, 0.01) → 0 (0, 0.01)	NaN%	-1.2% (-3.2%, 0.84%)
		Female	0 (0, 0.01) → 0 (0, 0)	NaN%	-3.96% (-5.29%, -2.6%)	0 (0, 0) → 0 (0, 0.01)	NaN%	0.21% (-1.87%, 2.32%)
		Male	0.02 (0, 0.07) → 0.01 (0, 0.01)	-50%	-7.03% (-8.31%, -5.73%)	0.01 (0, 0.01) → 0 (0, 0.01)	-100%	-1.62% (-3.61%, 0.4%)
	Italy	Both	0 (0, 0.02) → 0 (0, 0.01)	NaN%	-2.4% (-5.13%, 0.41%)	0 (0, 0.01) → 0 (0, 0)	NaN%	-8.44% (-10.14%, -6.7%)
		Female	0 (0, 0.01) → 0 (0, 0)	NaN%	-1.24% (-4.4%, 2.03%)	0 (0, 0) → 0 (0, 0)	NaN%	-7.75% (-9.4%, -6.08%)
		Male	0.01 (0, 0.03) → 0.01 (0, 0.01)	0%	-2.65% (-5.28%, 0.05%)	0.01 (0, 0.01) → 0 (0, 0.01)	-100%	-8.68% (-10.43%, -6.91%)
	Japan	Both	0 (0, 0) → 0 (0, 0)	NaN%	1.57% (-3.02%, 6.39%)	0 (0, 0) → 0 (0, 0)	NaN%	-0.35% (-1.01%, 0.32%)
		Female	0 (0, 0) → 0 (0, 0)	NaN%	0.74% (-3.56%, 5.23%)	0 (0, 0) → 0 (0, 0)	NaN%	2.14% (1.28%, 3.01%)
		Male	0 (0, 0) → 0 (0, 0)	NaN%	1.83% (-2.86%, 6.76%)	0 (0, 0) → 0 (0, 0)	NaN%	-1.33% (-1.93%, -0.73%)
	United Kingdom	Both	0.01 (0, 0.02) → 0 (0, 0.01)	-100%	-5.98% (-7.74%, -4.18%)	0 (0, 0.01) → 0 (0, 0)	NaN%	-6.64% (-8.14%, -5.11%)
		Female	0 (0, 0) → 0 (0, 0)	NaN%	1.02% (-0.71%, 2.79%)	0 (0, 0) → 0 (0, 0)	NaN%	-5.96% (-7.44%, -4.47%)
		Male	0.01 (0, 0.03) → 0 (0, 0.01)	-100%	-7.93% (-9.82%, -6%)	0 (0, 0.01) → 0 (0, 0)	NaN%	-6.96% (-8.63%, -5.27%)
	United States of America	Both	0.03 (0.01, 0.08) → 0 (0, 0)	-100%	-15.88% (-17.6%, -14.13%)	0 (0, 0) → 0 (0, 0)	NaN%	-1.8% (-3.93%, 0.38%)
		Female	0.01 (0, 0.02) → 0 (0, 0)	-100%	-12.2% (-13.35%, -11.03%)	0 (0, 0) → 0 (0, 0)	NaN%	-2.18% (-4.32%, 0.01%)
		Male	0.06 (0.02, 0.14) → 0 (0, 0.01)	-100%	-16.82% (-18.67%, -14.94%)	0 (0, 0.01) → 0 (0, 0.01)	NaN%	-1.68% (-3.8%, 0.49%)
HIV/AIDS resulting in other diseases	Canada	Both	2.18 (1.84, 2.49) → 0.69 (0.61, 0.76)	-68.3%	-7.64% (-9.18%, -6.09%)	0.69 (0.61, 0.76) → 0.3 (0.25, 0.34)	-56.5%	-7.59% (-8.68%, -6.48%)
		Female	0.33 (0.28, 0.38) → 0.32 (0.28, 0.36)	-3%	-1.79% (-3.15%, -0.42%)	0.32 (0.28, 0.36) → 0.15 (0.12, 0.18)	-53.1%	-6.94% (-7.54%, -6.34%)
		Male	4.05 (3.38, 4.66) → 1.08 (0.93, 1.2)	-73.3%	-8.54% (-10.15%, -6.89%)	1.08 (0.93, 1.2) → 0.45 (0.36, 0.53)	-58.3%	-7.81% (-9.08%, -6.52%)
	France	Both	2.76 (2.32, 3.46) → 0.59 (0.47, 0.73)	-78.6%	-10.22% (-12.16%, -8.23%)	0.59 (0.47, 0.73) → 0.32 (0.25, 0.4)	-45.8%	-5.98% (-6.68%, -5.28%)
		Female	0.78 (0.66, 1) → 0.34 (0.27, 0.43)	-56.4%	-7.4% (-9.42%, -5.34%)	0.34 (0.27, 0.43) → 0.19 (0.14, 0.25)	-44.1%	-5.42% (-5.97%, -4.88%)
		Male	4.76 (3.93, 5.98) → 0.85 (0.67, 1.08)	-82.1%	-10.9% (-12.83%, -8.92%)	0.85 (0.67, 1.08) → 0.46 (0.35, 0.59)	-45.9%	-6.17% (-6.97%, -5.37%)
	Germany	Both	1.18 (0.95, 1.46) → 0.49 (0.43, 0.54)	-58.5%	-7.47% (-9.29%, -5.61%)	0.49 (0.43, 0.54) → 0.27 (0.23, 0.3)	-44.9%	-4.5% (-5.94%, -3.03%)
		Female	0.35 (0.28, 0.44) → 0.23 (0.2, 0.27)	-34.3%	-4.95% (-6.57%, -3.31%)	0.23 (0.2, 0.27) → 0.15 (0.12, 0.17)	-34.8%	-3.11% (-4.66%, -1.52%)
		Male	1.99 (1.58, 2.5) → 0.74 (0.63, 0.83)	-62.8%	-8.04% (-9.94%, -6.1%)	0.74 (0.63, 0.83) → 0.39 (0.32, 0.45)	-47.3%	-4.91% (-6.33%, -3.47%)
	Italy	Both	2.74 (2.55, 2.95) → 1.15 (1.08, 1.21)	-58%	-7.84% (-9.89%, -5.74%)	1.15 (1.08, 1.21) → 0.58 (0.54, 0.61)	-49.6%	-6.55% (-7.03%, -6.07%)
		Female	0.97 (0.87, 1.06) → 0.55 (0.5, 0.59)	-43.3%	-6.71% (-8.86%, -4.51%)	0.55 (0.5, 0.59) → 0.29 (0.27, 0.31)	-47.3%	-5.78% (-6.13%, -5.44%)
		Male	4.52 (4.15, 4.9) → 1.78 (1.65, 1.89)	-60.6%	-8.08% (-10.13%, -5.98%)	1.78 (1.65, 1.89) → 0.88 (0.81, 0.94)	-50.6%	-6.83% (-7.44%, -6.22%)
	Japan	Both	0.02 (0.02, 0.03) → 0.08 (0.07, 0.1)	300%	5.9% (4.79%, 7.02%)	0.08 (0.07, 0.1) → 0.08 (0.06, 0.09)	0%	-0.45% (-1.01%, 0.11%)
		Female	0.01 (0.01, 0.02) → 0.04 (0.04, 0.05)	300%	5.19% (4.36%, 6.03%)	0.04 (0.04, 0.05) → 0.05 (0.04, 0.06)	25%	1.98% (1.32%, 2.63%)
		Male	0.04 (0.03, 0.04) → 0.12 (0.1, 0.15)	200%	6.12% (4.91%, 7.34%)	0.12 (0.1, 0.15) → 0.1 (0.09, 0.12)	-16.7%	-1.45% (-2.01%, -0.87%)
	United Kingdom	Both	0.61 (0.52, 0.7) → 0.37 (0.33, 0.41)	-39.3%	-4.98% (-7.06%, -2.86%)	0.37 (0.33, 0.41) → 0.26 (0.24, 0.28)	-29.7%	-2.85% (-3.66%, -2.04%)
		Female	0.11 (0.09, 0.12) → 0.26 (0.23, 0.29)	136.4%	2.44% (0.58%, 4.34%)	0.26 (0.23, 0.29) → 0.19 (0.17, 0.2)	-26.9%	-2.13% (-3.56%, -0.67%)
		Male	1.12 (0.95, 1.29) → 0.49 (0.43, 0.54)	-56.2%	-6.95% (-9.15%, -4.7%)	0.49 (0.43, 0.54) → 0.33 (0.3, 0.36)	-32.7%	-3.2% (-3.87%, -2.51%)
	United States of America	Both	9.2 (8.81, 9.59) → 2.49 (2.4, 2.56)	-72.9%	-7.99% (-9.19%, -6.77%)	2.49 (2.4, 2.56) → 1.42 (1.35, 1.48)	-43%	-4.99% (-5.49%, -4.48%)
		Female	2.26 (2.15, 2.36) → 1.46 (1.4, 1.53)	-35.4%	-3.64% (-4.98%, -2.28%)	1.46 (1.4, 1.53) → 0.79 (0.75, 0.83)	-45.9%	-5.44% (-5.92%, -4.97%)
		Male	16.34 (15.57, 17.08) → 3.56 (3.4, 3.68)	-78.2%	-9.1% (-10.38%, -7.8%)	3.56 (3.4, 3.68) → 2.07 (1.95, 2.18)	-41.9%	-4.83% (-5.34%, -4.31%)

### 3.4 Age-standardized prevalence rate: chronic burden accumulation driven by improved survival

Against the backdrop of declining ASIR and ASMR, ASPR reflected changes in the burden among surviving populations. From 1990 to 2010, ASPR increased markedly in most countries; for example, France rose from 32.79 to 45.45 (AAPC 2.16, 95% CI: 2.00 to 2.49%), while Japan increased from 1.48 to 5.04 (AAPC 5.94, 95% CI: 4.92 to 6.97%), reflecting survival accumulation associated with ART.

During 2010–2021, ASPR trends diverged across countries. In the United States, ASPR declined from 105.61 to 78.74 (AAPC −2.60, 95% CI: −2.86 to −2.34%), whereas Japan continued to increase (AAPC 0.64, 95% CI: 0.28 to 1.00%). France and Germany maintained moderate upward trends (e.g., France AAPC 1.73, 95% CI: 1.65 to 1.81%).

Sex differences indicated that ASPR increased more rapidly in females in some countries; for example, UK females rose from 6.79 to 20.14 (AAPC 3.85, 95% CI: 2.29 to 5.44%). Subtype analysis showed that ASPR for drug-susceptible tuberculosis declined substantially (e.g., Germany from 3.08 to 0.56, AAPC −8.85, 95% CI: −9.56 to −8.14%), while extensively drug-resistant tuberculosis remained at extremely low levels (Table 3).

**Table 3:** Results of ASPR in G7 countries.

Cause	Country	Sex	Value_ (1990-2010)	PC	AAPC_UI_ (1990-2010)	Value_ (2010-2021)	PC	AAPC_UI_ (2010-2021)
HIV/AIDS	Canada	Both	117.99 (73.07, 171.87) → 138.2 (99.16, 180.2)	17.1%	0.62% (0.49%, 0.75%)	138.2 (99.16, 180.2) → 173.36 (128.84, 226.95)	25.4%	2.25% (2%, 2.49%)
		Female	21.08 (9.37, 37.28) → 71.69 (42.39, 103.05)	240.1%	6.25% (5.63%, 6.87%)	71.69 (42.39, 103.05) → 96.67 (62.11, 131.62)	34.8%	2.84% (2.75%, 2.94%)
		Male	215.6 (136.95, 309.83) → 207.41 (154.48, 264.91)	-3.8%	-0.43% (-0.57%, -0.29%)	207.41 (154.48, 264.91) → 253.14 (188.41, 334.32)	22%	2.02% (1.72%, 2.32%)
	France	Both	142.53 (136.58, 148.25) → 234.67 (218.53, 251.89)	64.6%	2.85% (2.55%, 3.14%)	234.67 (218.53, 251.89) → 283.64 (262.95, 304.58)	20.9%	1.73% (1.65%, 1.81%)
		Female	52.77 (44.6, 61.2) → 109.89 (96.61, 125.75)	108.2%	4.12% (3.81%, 4.43%)	109.89 (96.61, 125.75) → 141.18 (122.55, 157.71)	28.5%	2.3% (2.16%, 2.43%)
		Male	234.57 (221.38, 248.91) → 365.11 (333.85, 397.8)	55.7%	2.54% (2.26%, 2.83%)	365.11 (333.85, 397.8) → 434.06 (392.93, 474.47)	18.9%	1.58% (1.51%, 1.64%)
	Germany	Both	58.91 (43.04, 72.94) → 69.06 (55.15, 85.59)	17.2%	0.62% (0.35%, 0.88%)	69.06 (55.15, 85.59) → 79.49 (62.69, 100.29)	15.1%	1.44% (1.29%, 1.6%)
		Female	20.65 (14.07, 28.75) → 38.11 (26.21, 53.49)	84.6%	2.95% (2.62%, 3.28%)	38.11 (26.21, 53.49) → 47.86 (32.3, 67.89)	25.6%	2.24% (1.88%, 2.61%)
		Male	95.91 (70.09, 121.45) → 100.22 (80.41, 130.05)	4.5%	0.05% (-0.18%, 0.27%)	100.22 (80.41, 130.05) → 111.83 (83.91, 144.07)	11.6%	1.15% (1.04%, 1.25%)
	Italy	Both	141.34 (107.29, 173.55) → 185.47 (153.94, 224.2)	31.2%	1.17% (1.03%, 1.31%)	185.47 (153.94, 224.2) → 180.67 (140.87, 219.88)	-2.6%	-0.29% (-0.37%, -0.22%)
		Female	58.65 (42.26, 78.07) → 107.45 (84.05, 142.34)	83.2%	2.95% (2.8%, 3.11%)	107.45 (84.05, 142.34) → 113.71 (83.79, 150.49)	5.8%	0.47% (0.3%, 0.64%)
		Male	224.62 (168.41, 278.5) → 266.51 (213.21, 322.96)	18.6%	0.63% (0.49%, 0.77%)	266.51 (213.21, 322.96) → 250.63 (196.65, 308.23)	-6%	-0.62% (-0.68%, -0.56%)
	Japan	Both	2.84 (1.5, 4.53) → 14.14 (8.93, 21)	397.9%	8.09% (7.58%, 8.61%)	14.14 (8.93, 21) → 26.18 (16.71, 37.04)	85.1%	5.73% (5.29%, 6.17%)
		Female	1.42 (0.75, 2.25) → 8 (5.02, 11.99)	463.4%	8.71% (8.19%, 9.22%)	8 (5.02, 11.99) → 15.47 (9.9, 21.94)	93.4%	6.11% (5.56%, 6.65%)
		Male	4.26 (2.28, 6.87) → 20.37 (12.76, 29.86)	378.2%	7.89% (7.38%, 8.41%)	20.37 (12.76, 29.86) → 37.11 (23.59, 52.63)	82.2%	5.6% (5.2%, 5.99%)
	United Kingdom	Both	33.31 (23.77, 43.47) → 123.35 (94.15, 144.56)	270.3%	7.56% (7.06%, 8.07%)	123.35 (94.15, 144.56) → 148.55 (108.88, 180.26)	20.4%	1.73% (1.66%, 1.8%)
		Female	9.97 (6.67, 13.59) → 81.54 (60.11, 98.95)	717.9%	12.45% (11.01%, 13.91%)	81.54 (60.11, 98.95) → 99.81 (73.27, 123.45)	22.4%	1.9% (1.83%, 1.97%)
		Male	56.99 (40.85, 73.91) → 166.38 (129.76, 198.92)	191.9%	6.01% (5.7%, 6.31%)	166.38 (129.76, 198.92) → 199.87 (148.63, 241.51)	20.1%	1.7% (1.63%, 1.77%)
	United States of America	Both	307.14 (201.67, 425.23) → 355.15 (239.11, 480.23)	15.6%	0.59% (0.46%, 0.71%)	355.15 (239.11, 480.23) → 417.3 (250.11, 591.6)	17.5%	1.54% (1.49%, 1.59%)
		Female	107.51 (65.84, 156.92) → 189.41 (126.06, 260.76)	76.2%	2.78% (2.65%, 2.91%)	189.41 (126.06, 260.76) → 238.66 (150.13, 349.28)	26%	2.19% (2.14%, 2.24%)
		Male	509.64 (340.17, 706.8) → 526.96 (341.34, 725.88)	3.4%	0% (-0.15%, 0.15%)	526.96 (341.34, 725.88) → 601.13 (365.94, 839.3)	14.1%	1.26% (1.22%, 1.31%)
HIV/AIDS - Drug-susceptible tuberculosis	Canada	Both	1.47 (1.22, 1.75) → 0.45 (0.38, 0.53)	-69.4%	-7.25% (-8.16%, -6.34%)	0.45 (0.38, 0.53) → 0.33 (0.28, 0.39)	-26.7%	-2.79% (-3.84%, -1.73%)
		Female	0.26 (0.22, 0.31) → 0.23 (0.2, 0.27)	-11.5%	-2.26% (-3.34%, -1.16%)	0.23 (0.2, 0.27) → 0.18 (0.16, 0.22)	-21.7%	-2.24% (-3.16%, -1.31%)
		Male	2.69 (2.23, 3.19) → 0.68 (0.57, 0.8)	-74.7%	-8.2% (-9.12%, -7.27%)	0.68 (0.57, 0.8) → 0.48 (0.41, 0.56)	-29.4%	-3% (-4.1%, -1.89%)
	France	Both	6.86 (5.71, 8.18) → 0.97 (0.82, 1.12)	-85.9%	-10.57% (-11.49%, -9.64%)	0.97 (0.82, 1.12) → 0.43 (0.37, 0.5)	-55.7%	-7.08% (-7.63%, -6.52%)
		Female	2.53 (2.11, 3.02) → 0.45 (0.39, 0.52)	-82.2%	-9.51% (-10.42%, -8.59%)	0.45 (0.39, 0.52) → 0.21 (0.18, 0.25)	-53.3%	-6.55% (-7.06%, -6.04%)
		Male	11.3 (9.4, 13.48) → 1.51 (1.28, 1.74)	-86.6%	-10.82% (-11.75%, -9.88%)	1.51 (1.28, 1.74) → 0.66 (0.56, 0.76)	-56.3%	-7.22% (-7.79%, -6.64%)
	Germany	Both	2.43 (2.05, 2.85) → 0.36 (0.3, 0.41)	-85.2%	-10.98% (-12.04%, -9.91%)	0.36 (0.3, 0.41) → 0.22 (0.18, 0.25)	-38.9%	-4.38% (-4.45%, -4.3%)
		Female	0.85 (0.71, 0.99) → 0.2 (0.17, 0.23)	-76.5%	-8.93% (-10.03%, -7.82%)	0.2 (0.17, 0.23) → 0.13 (0.11, 0.15)	-35%	-3.63% (-3.93%, -3.33%)
		Male	3.96 (3.35, 4.64) → 0.52 (0.44, 0.6)	-86.9%	-11.48% (-12.52%, -10.43%)	0.52 (0.44, 0.6) → 0.31 (0.26, 0.36)	-40.4%	-4.66% (-4.68%, -4.63%)

	Italy	Both	0.86 (0.72, 1.02) → 0.21 (0.17, 0.25)	-75.6%	-9.14% (-10.42%, -7.84%)	0.21 (0.17, 0.25) → 0.1 (0.09, 0.12)	-52.4%	-6.55% (-6.85%, -6.25%)
		Female	0.36 (0.3, 0.43) → 0.12 (0.1, 0.14)	-66.7%	-7.55% (-8.83%, -6.25%)	0.12 (0.1, 0.14) → 0.07 (0.05, 0.08)	-41.7%	-5.87% (-6.19%, -5.54%)
		Male	1.37 (1.15, 1.62) → 0.3 (0.25, 0.35)	-78.1%	-9.64% (-10.93%, -8.33%)	0.3 (0.25, 0.35) → 0.14 (0.12, 0.16)	-53.3%	-6.85% (-7.15%, -6.55%)
	Japan	Both	0.16 (0.13, 0.18) → 0.15 (0.12, 0.18)	-6.3%	-1.2% (-2.41%, 0.02%)	0.15 (0.12, 0.18) → 0.09 (0.07, 0.11)	-40%	-4.26% (-4.55%, -3.96%)
		Female	0.08 (0.07, 0.09) → 0.08 (0.07, 0.1)	0%	-0.7% (-1.91%, 0.52%)	0.08 (0.07, 0.1) → 0.05 (0.04, 0.06)	-37.5%	-3.9% (-4.13%, -3.68%)
		Male	0.24 (0.2, 0.28) → 0.22 (0.17, 0.26)	-8.3%	-1.37% (-2.58%, -0.14%)	0.22 (0.17, 0.26) → 0.13 (0.1, 0.16)	-40.9%	-4.38% (-4.71%, -4.06%)
	United Kingdom	Both	1.1 (0.93, 1.3) → 0.41 (0.35, 0.47)	-62.7%	-6.16% (-6.95%, -5.37%)	0.41 (0.35, 0.47) → 0.2 (0.17, 0.23)	-51.2%	-6.3% (-6.48%, -6.13%)
		Female	0.32 (0.27, 0.38) → 0.28 (0.24, 0.32)	-12.5%	-1.67% (-2.52%, -0.82%)	0.28 (0.24, 0.32) → 0.14 (0.12, 0.16)	-50%	-6.25% (-6.41%, -6.08%)
		Male	1.9 (1.6, 2.24) → 0.54 (0.46, 0.62)	-71.6%	-7.64% (-8.6%, -6.67%)	0.54 (0.46, 0.62) → 0.27 (0.23, 0.31)	-50%	-6.29% (-6.46%, -6.11%)
	United States of America	Both	1.49 (1.24, 1.77) → 0.29 (0.25, 0.34)	-80.5%	-9.11% (-10.12%, -8.08%)	0.29 (0.25, 0.34) → 0.23 (0.2, 0.27)	-20.7%	-1.88% (-2.6%, -1.15%)
		Female	0.51 (0.42, 0.6) → 0.15 (0.13, 0.18)	-70.6%	-7.16% (-8.28%, -6.03%)	0.15 (0.13, 0.18) → 0.13 (0.11, 0.15)	-13.3%	-1.28% (-2.05%, -0.51%)
Male		2.49 (2.06, 2.96) → 0.44 (0.37, 0.51)	-82.3%	-9.61% (-10.61%, -8.6%)	0.44 (0.37, 0.51) → 0.34 (0.29, 0.4)	-22.7%	-2.13% (-2.82%, -1.42%)
HIV/AIDS - Extensively drug-resistant tuberculosis	Canada	Both	0 (0, 0) → 0 (0, 0)	NaN%	29.41% (3.51%, 61.8%)	0 (0, 0) → 0 (0, 0)	NaN%	2.18% (1.21%, 3.16%)
		Female	0 (0, 0) → 0 (0, 0)	NaN%	32.87% (8.69%, 62.44%)	0 (0, 0) → 0 (0, 0)	NaN%	2.74% (1.91%, 3.58%)
		Male	0 (0, 0) → 0 (0, 0)	NaN%	29.12% (2.33%, 62.92%)	0 (0, 0) → 0 (0, 0)	NaN%	1.96% (0.93%, 2.99%)
	France	Both	0 (0, 0) → 0 (0, 0)	NaN%	33.84% (5.66%, 69.52%)	0 (0, 0) → 0 (0, 0)	NaN%	-1.62% (-1.76%, -1.48%)
		Female	0 (0, 0) → 0 (0, 0)	NaN%	33.57% (7.02%, 66.71%)	0 (0, 0) → 0 (0, 0)	NaN%	-1.1% (-1.21%, -0.99%)
		Male	0 (0, 0) → 0 (0, 0)	NaN%	34.34% (5.25%, 71.46%)	0 (0, 0) → 0 (0, 0)	NaN%	-1.76% (-1.91%, -1.61%)
	Germany	Both	0 (0, 0) → 0 (0, 0)	NaN%	32.18% (5.74%, 65.23%)	0 (0, 0) → 0 (0, 0)	NaN%	4.58% (2.96%, 6.22%)
		Female	0 (0, 0) → 0 (0, 0)	NaN%	33.23% (8.3%, 63.89%)	0 (0, 0) → 0 (0, 0)	NaN%	5.28% (3.43%, 7.17%)
		Male	0 (0, 0) → 0 (0, 0)	NaN%	32.3% (5.03%, 66.64%)	0 (0, 0) → 0 (0, 0)	NaN%	4.31% (2.79%, 5.86%)
	Italy	Both	0 (0, 0) → 0 (0, 0)	NaN%	35.15% (7.55%, 69.83%)	0 (0, 0) → 0 (0, 0)	NaN%	-5.05% (-5.39%, -4.71%)
		Female	0 (0, 0) → 0 (0, 0)	NaN%	35.91% (9.56%, 68.59%)	0 (0, 0) → 0 (0, 0)	NaN%	-4.39% (-4.68%, -4.1%)
		Male	0 (0, 0) → 0 (0, 0)	NaN%	35.23% (6.86%, 71.14%)	0 (0, 0) → 0 (0, 0)	NaN%	-5.35% (-5.7%, -5%)
	Japan	Both	0 (0, 0) → 0 (0, 0)	NaN%	28.22% (4.54%, 57.26%)	0 (0, 0) → 0 (0, 0)	NaN%	1.82% (1.57%, 2.07%)
		Female	0 (0, 0) → 0 (0, 0)	NaN%	27.8% (5.29%, 55.12%)	0 (0, 0) → 0 (0, 0)	NaN%	2.19% (1.88%, 2.51%)
		Male	0 (0, 0) → 0 (0, 0)	NaN%	28.65% (4.25%, 58.77%)	0 (0, 0) → 0 (0, 0)	NaN%	1.69% (1.46%, 1.91%)

	United Kingdom	Both	0 (0, 0) → 0 (0, 0)	NaN%	29% (3.83%, 60.27%)	0 (0, 0) → 0 (0, 0)	NaN%	-2.01% (-2.73%, -1.28%)
		Female	0 (0, 0) → 0 (0, 0)	NaN%	33.04% (8.85%, 62.6%)	0 (0, 0) → 0 (0, 0)	NaN%	-1.99% (-2.7%, -1.28%)
		Male	0 (0, 0) → 0 (0, 0)	NaN%	27.84% (2.05%, 60.15%)	0 (0, 0) → 0 (0, 0)	NaN%	-1.96% (-2.69%, -1.24%)
	United States of America	Both	0 (0, 0) → 0 (0, 0)	NaN%	22.62% (-3%, 55%)	0 (0, 0) → 0 (0, 0)	NaN%	3.24% (2.47%, 4.02%)
		Female	0 (0, 0) → 0 (0, 0)	NaN%	23.13% (-1.11%, 53.33%)	0 (0, 0) → 0 (0, 0)	NaN%	3.83% (3%, 4.66%)
Male		0 (0, 0) → 0 (0, 0)	NaN%	22.83% (-3.57%, 56.46%)	0 (0, 0) → 0 (0, 0)	NaN%	3% (2.25%, 3.75%)
HIV/AIDS - Multidrug-resistant tuberculosis without extensive drug resistance	Canada	Both	0.01 (0, 0.04) → 0.01 (0, 0.01)	0%	-5.85% (-7.22%, -4.45%)	0.01 (0, 0.01) → 0.01 (0, 0.01)	0%	-2.44% (-3.53%, -1.33%)
		Female	0 (0, 0.01) → 0 (0, 0)	NaN%	-1.3% (-2.93%, 0.36%)	0 (0, 0) → 0 (0, 0.01)	NaN%	-1.95% (-2.92%, -0.96%)
		Male	0.03 (0.01, 0.07) → 0.01 (0.01, 0.01)	-66.7%	-6.78% (-8.16%, -5.38%)	0.01 (0.01, 0.01) → 0.01 (0, 0.02)	0%	-2.63% (-3.77%, -1.47%)
	France	Both	0.04 (0.01, 0.12) → 0.02 (0.01, 0.03)	-50%	-4.26% (-5.51%, -2.98%)	0.02 (0.01, 0.03) → 0.01 (0.01, 0.02)	-50%	-6.11% (-6.42%, -5.8%)
		Female	0.01 (0, 0.05) → 0.01 (0.01, 0.01)	0%	-3.23% (-4.51%, -1.93%)	0.01 (0.01, 0.01) → 0 (0, 0.01)	-100%	-5.63% (-5.91%, -5.35%)
		Male	0.06 (0.01, 0.2) → 0.03 (0.02, 0.04)	-50%	-4.51% (-5.77%, -3.23%)	0.03 (0.02, 0.04) → 0.01 (0.01, 0.03)	-66.7%	-6.25% (-6.57%, -5.92%)
	Germany	Both	0.02 (0, 0.06) → 0.01 (0.01, 0.01)	-50%	-4.42% (-5.48%, -3.36%)	0.01 (0.01, 0.01) → 0.01 (0, 0.03)	0%	-0.12% (-1.48%, 1.27%)
		Female	0.01 (0, 0.02) → 0.01 (0, 0.01)	0%	-2.32% (-3.4%, -1.23%)	0.01 (0, 0.01) → 0.01 (0, 0.02)	0%	0.64% (-0.96%, 2.27%)
		Male	0.03 (0.01, 0.1) → 0.02 (0.01, 0.02)	-33.3%	-4.95% (-6.01%, -3.89%)	0.02 (0.01, 0.02) → 0.01 (0, 0.04)	-50%	-0.4% (-1.67%, 0.89%)
	Italy	Both	0.01 (0, 0.03) → 0.01 (0.01, 0.01)	0%	-1.13% (-4.23%, 2.06%)	0.01 (0.01, 0.01) → 0 (0, 0.01)	-100%	-9.4% (-9.86%, -8.94%)
		Female	0 (0, 0.01) → 0.01 (0, 0.01)	Inf%	0.48% (-2.71%, 3.77%)	0.01 (0, 0.01) → 0 (0, 0)	-100%	-8.76% (-9.16%, -8.35%)
		Male	0.01 (0, 0.05) → 0.01 (0.01, 0.02)	0%	-1.64% (-4.73%, 1.54%)	0.01 (0.01, 0.02) → 0 (0, 0.01)	-100%	-9.69% (-10.16%, -9.22%)
	Japan	Both	0 (0, 0) → 0 (0, 0)	NaN%	-3.29% (-7.24%, 0.83%)	0 (0, 0) → 0 (0, 0)	NaN%	-2.87% (-3.04%, -2.7%)
		Female	0 (0, 0) → 0 (0, 0)	NaN%	-2.81% (-6.78%, 1.33%)	0 (0, 0) → 0 (0, 0)	NaN%	-2.52% (-2.7%, -2.34%)
		Male	0 (0, 0) → 0 (0, 0)	NaN%	-3.45% (-7.4%, 0.67%)	0 (0, 0) → 0 (0, 0)	NaN%	-2.99% (-3.17%, -2.82%)
	United Kingdom	Both	0.01 (0, 0.03) → 0.01 (0, 0.01)	0%	-5.91% (-7.56%, -4.23%)	0.01 (0, 0.01) → 0 (0, 0.01)	-100%	-6.61% (-7.13%, -6.07%)
		Female	0 (0, 0.01) → 0 (0, 0.01)	NaN%	-1.59% (-2.98%, -0.18%)	0 (0, 0.01) → 0 (0, 0)	NaN%	-6.56% (-7.08%, -6.03%)
		Male	0.02 (0.01, 0.05) → 0.01 (0.01, 0.01)	-50%	-7.38% (-9.2%, -5.52%)	0.01 (0.01, 0.01) → 0 (0, 0.01)	-100%	-6.58% (-7.12%, -6.05%)
	United States of America	Both	0.05 (0.02, 0.09) → 0 (0, 0.01)	-100%	-13.03% (-14.14%, -11.91%)	0 (0, 0.01) → 0 (0, 0.01)	NaN%	-1.46% (-2.39%, -0.53%)
		Female	0.02 (0.01, 0.03) → 0 (0, 0)	-100%	-11.34% (-12.41%, -10.27%)	0 (0, 0) → 0 (0, 0.01)	NaN%	-0.91% (-1.89%, 0.07%)
		Male	0.09 (0.04, 0.15) → 0.01 (0.01, 0.01)	-88.9%	-13.48% (-14.61%, -12.34%)	0.01 (0.01, 0.01) → 0.01 (0, 0.01)	0%	-1.7% (-2.6%, -0.78%)
HIV/AIDS resulting in other diseases	Canada	Both	116.5 (71.52, 170.43) → 137.74 (98.71, 179.77)	18.2%	0.68% (0.55%, 0.81%)	137.74 (98.71, 179.77) → 173.03 (128.47, 226.62)	25.6%	2.26% (2.02%, 2.5%)
		Female	20.81 (9.09, 37.01) → 71.45 (42.17, 102.81)	243.3%	6.32% (5.7%, 6.94%)	71.45 (42.17, 102.81) → 96.49 (61.93, 131.45)	35%	2.86% (2.76%, 2.95%)
		Male	212.89 (134.1, 307.2) → 206.72 (153.8, 264.22)	-2.9%	-0.37% (-0.5%, -0.23%)	206.72 (153.8, 264.22) → 252.65 (187.81, 333.83)	22.2%	2.03% (1.73%, 2.33%)
	France	Both	135.63 (129.99, 141.39) → 233.67 (217.54, 250.91)	72.3%	3.14% (2.84%, 3.44%)	233.67 (217.54, 250.91) → 283.2 (262.54, 304.12)	21.2%	1.75% (1.67%, 1.84%)
		Female	50.23 (42.04, 58.61) → 109.43 (96.13, 125.26)	117.9%	4.42% (4.1%, 4.74%)	109.43 (96.13, 125.26) → 140.96 (122.36, 157.53)	28.8%	2.32% (2.18%, 2.46%)
		Male	223.21 (210.59, 237.44) → 363.57 (332.18, 396.3)	62.9%	2.83% (2.54%, 3.12%)	363.57 (332.18, 396.3) → 433.39 (392.29, 473.88)	19.2%	1.6% (1.53%, 1.67%)
	Germany	Both	56.46 (40.47, 70.68) → 68.7 (54.75, 85.22)	21.7%	0.83% (0.59%, 1.07%)	68.7 (54.75, 85.22) → 79.26 (62.44, 100.05)	15.4%	1.47% (1.31%, 1.62%)
		Female	19.8 (13.18, 27.99) → 37.91 (26.02, 53.29)	91.5%	3.17% (2.87%, 3.47%)	37.91 (26.02, 53.29) → 47.72 (32.16, 67.74)	25.9%	2.27% (1.9%, 2.63%)
		Male	91.92 (66.06, 117.39) → 99.69 (79.86, 129.51)	8.5%	0.26% (0.06%, 0.46%)	99.69 (79.86, 129.51) → 111.51 (83.57, 143.74)	11.9%	1.17% (1.06%, 1.28%)
	Italy	Both	140.47 (106.35, 172.78) → 185.25 (153.71, 223.98)	31.9%	1.21% (1.07%, 1.35%)	185.25 (153.71, 223.98) → 180.56 (140.76, 219.78)	-2.5%	-0.29% (-0.36%, -0.21%)
		Female	58.29 (41.92, 77.73) → 107.32 (83.91, 142.21)	84.1%	2.99% (2.84%, 3.15%)	107.32 (83.91, 142.21) → 113.64 (83.72, 150.42)	5.9%	0.48% (0.31%, 0.64%)
		Male	223.24 (167, 277.11) → 266.2 (212.91, 322.63)	19.2%	0.67% (0.53%, 0.8%)	266.2 (212.91, 322.63) → 250.48 (196.5, 308.07)	-5.9%	-0.62% (-0.68%, -0.56%)
	Japan	Both	2.68 (1.34, 4.37) → 13.99 (8.77, 20.85)	422%	8.41% (7.89%, 8.93%)	13.99 (8.77, 20.85) → 26.09 (16.61, 36.95)	86.5%	5.79% (5.35%, 6.24%)
		Female	1.34 (0.67, 2.17) → 7.91 (4.93, 11.9)	490.3%	9.02% (8.5%, 9.55%)	7.91 (4.93, 11.9) → 15.41 (9.85, 21.89)	94.8%	6.17% (5.62%, 6.73%)
		Male	4.03 (2.02, 6.63) → 20.15 (12.53, 29.66)	400%	8.2% (7.69%, 8.72%)	20.15 (12.53, 29.66) → 36.98 (23.46, 52.5)	83.5%	5.66% (5.26%, 6.07%)
	United Kingdom	Both	32.2 (22.64, 42.28) → 122.93 (93.75, 144.18)	281.8%	7.76% (7.22%, 8.29%)	122.93 (93.75, 144.18) → 148.34 (108.67, 180.07)	20.7%	1.75% (1.68%, 1.82%)
		Female	9.64 (6.34, 13.28) → 81.26 (59.86, 98.69)	742.9%	12.66% (11.18%, 14.16%)	81.26 (59.86, 98.69) → 99.66 (73.13, 123.32)	22.6%	1.92% (1.85%, 1.99%)
		Male	55.07 (38.76, 72.2) → 165.84 (129.19, 198.34)	201.1%	6.2% (5.88%, 6.52%)	165.84 (129.19, 198.34) → 199.6 (148.37, 241.25)	20.4%	1.72% (1.65%, 1.79%)
	United States of America	Both	305.6 (199.94, 423.73) → 354.85 (238.8, 479.94)	16.1%	0.61% (0.49%, 0.73%)	354.85 (238.8, 479.94) → 417.07 (249.87, 591.38)	17.5%	1.54% (1.49%, 1.59%)
		Female	106.99 (65.3, 156.48) → 189.25 (125.9, 260.61)	76.9%	2.81% (2.68%, 2.94%)	189.25 (125.9, 260.61) → 238.53 (150, 349.15)	26%	2.19% (2.14%, 2.24%)
		Male	507.06 (337.15, 704.29) → 526.52 (340.89, 725.48)	3.8%	0.03% (-0.12%, 0.18%)	526.52 (340.89, 725.48) → 600.78 (365.57, 838.93)	14.1%	1.26% (1.22%, 1.31%)

### 3.5 Disability-adjusted life years: resurgence of comprehensive disease burden

As a composite measure of incidence, mortality, and survival quality, DALYs integrated the overall disease burden. From 1990 to 2010, DALYs increased substantially in most countries; for example, the United States rose from 1552.82 to 3333.88 (AAPC 2.68, 95% CI: 2.09 to 3.27%), while Japan increased sharply from 11.39 to 135.12 (AAPC 13.26, 95% CI: 13.04 to 13.47%), primarily driven by the growing number of surviving individuals.

During 2010–2021, DALYs continued to increase in most countries. For instance, France increased from 913.11 to 1254.35 (AAPC 3.06, 95% CI: 2.80 to 3.31%), and the United States showed a pronounced rise (AAPC 7.07, 95% CI: 6.84 to 7.30%). Japan maintained a rapid upward trend (AAPC 11.32, 95% CI: 10.99 to 11.64%).

Sex-stratified analysis showed substantially higher DALYs in males; for example, male DALYs in the United States increased from 1085.01 to 5,577. Among subtypes, DALYs attributable to drug-susceptible tuberculosis declined (e.g., Canada from 14.79 to 7.07, AAPC −4.27, 95% CI: −4.69 to −3.84%), whereas “other diseases resulting from HIV/AIDS” became the primary contributor to DALYs (Tables 4, 5).

**Table 4:** Results of DALYS in G7 countries.

Cause	Country	Sex	Value_ (1990-2010)	PC	AAPC_UI_ (1990-2010)	Value_ (2010-2021)	PC	AAPC_UI_ (2010-2021)
HIV/AIDS	Canada	Both	141.45 (127.45, 156.07) → 49.53 (41.63, 61.42)	-65%	-7.1% (-8.45%, -5.73%)	49.53 (41.63, 61.42) → 31.36 (25.14, 40.71)	-36.7%	-4.13% (-5.06%, -3.2%)
		Female	22.19 (19.56, 24.94) → 24.73 (19.76, 31.07)	11.4%	-0.89% (-2.08%, 0.31%)	24.73 (19.76, 31.07) → 17.7 (12.7, 24.33)	-28.4%	-3.14% (-3.69%, -2.59%)
		Male	261.76 (234.04, 290.32) → 75.25 (62.96, 94.71)	-71.3%	-8.1% (-9.53%, -6.65%)	75.25 (62.96, 94.71) → 45.51 (36.37, 59.07)	-39.5%	-4.51% (-5.57%, -3.43%)
	France	Both	249.9 (227.57, 273.48) → 61.48 (54.2, 69.74)	-75.4%	-9.47% (-11.14%, -7.77%)	61.48 (54.2, 69.74) → 42.58 (35.72, 51.13)	-30.7%	-3.31% (-3.6%, -3.02%)
		Female	76.02 (69.6, 83.91) → 33.53 (29.7, 38.08)	-55.9%	-7.12% (-8.87%, -5.33%)	33.53 (29.7, 38.08) → 23.5 (18.62, 28.25)	-29.9%	-3.39% (-3.67%, -3.11%)
		Male	424.04 (379.81, 470.48) → 90.67 (78.21, 104.41)	-78.6%	-10.04% (-11.71%, -8.33%)	90.67 (78.21, 104.41) → 62.77 (51.19, 76.9)	-30.8%	-3.24% (-3.55%, -2.93%)
	Germany	Both	94.37 (85.85, 103.21) → 31.92 (28.22, 35.72)	-66.2%	-7.98% (-9.64%, -6.3%)	31.92 (28.22, 35.72) → 19.39 (16.09, 22.95)	-39.3%	-3.94% (-4.84%, -3.03%)
		Female	30.21 (27.28, 33.58) → 17.23 (14.9, 19.88)	-43%	-5.36% (-6.78%, -3.92%)	17.23 (14.9, 19.88) → 12.48 (9.77, 15.57)	-27.6%	-2.16% (-3.16%, -1.15%)
		Male	156.4 (141.18, 174.57) → 46.51 (40.2, 53.04)	-70.3%	-8.65% (-10.39%, -6.87%)	46.51 (40.2, 53.04) → 26.4 (22.15, 31.7)	-43.2%	-4.62% (-5.5%, -3.73%)
	Italy	Both	179.13 (168.08, 191.09) → 71.82 (65.37, 81.8)	-59.9%	-7.92% (-9.83%, -5.96%)	71.82 (65.37, 81.8) → 40.57 (35.69, 47.39)	-43.5%	-5.24% (-5.48%, -4.99%)
		Female	65.81 (60.02, 71.41) → 37.82 (33.54, 43.53)	-42.5%	-6.48% (-8.49%, -4.42%)	37.82 (33.54, 43.53) → 23.59 (20.36, 28.06)	-37.6%	-4.12% (-4.54%, -3.7%)
		Male	292.28 (269.53, 313.66) → 106.91 (96.24, 123.79)	-63.4%	-8.29% (-10.2%, -6.34%)	106.91 (96.24, 123.79) → 58.02 (51.04, 67.72)	-45.7%	-5.69% (-6%, -5.38%)
	Japan	Both	2.52 (2.36, 2.71) → 6.97 (6.31, 7.79)	176.6%	4.8% (3.65%, 5.95%)	6.97 (6.31, 7.79) → 6.8 (5.89, 8.05)	-2.4%	0.02% (-0.16%, 0.2%)
		Female	1.45 (1.34, 1.57) → 3.68 (3.28, 4.13)	153.8%	4.35% (3.42%, 5.28%)	3.68 (3.28, 4.13) → 4.29 (3.72, 5.09)	16.6%	1.84% (1.5%, 2.18%)
		Male	3.59 (3.34, 3.89) → 10.25 (9.29, 11.32)	185.5%	4.95% (3.73%, 6.19%)	10.25 (9.29, 11.32) → 9.3 (8.03, 10.99)	-9.3%	-0.72% (-0.86%, -0.59%)
	United Kingdom	Both	45.15 (42.91, 47.54) → 32.09 (28.86, 36.04)	-28.9%	-3.7% (-5.32%, -2.05%)	32.09 (28.86, 36.04) → 25.45 (21.57, 30.28)	-20.7%	-1.77% (-2.28%, -1.25%)
		Female	9.07 (8.51, 9.7) → 23.29 (20.68, 26.51)	156.8%	3.3% (1.84%, 4.78%)	23.29 (20.68, 26.51) → 18.61 (15.8, 22.17)	-20.1%	-1.43% (-2.35%, -0.5%)
		Male	81.54 (77.11, 86.08) → 41.15 (36.87, 46.37)	-49.5%	-5.71% (-7.5%, -3.89%)	41.15 (36.87, 46.37) → 32.66 (27.36, 39.3)	-20.6%	-1.9% (-2.32%, -1.48%)
	United States of America	Both	522.14 (502.37, 544.71) → 140.93 (127.99, 156.77)	-73%	-8.08% (-9.25%, -6.89%)	140.93 (127.99, 156.77) → 93.2 (79.11, 110.27)	-33.9%	-3.7% (-4.24%, -3.16%)
		Female	137.03 (131.03, 142.85) → 85.3 (78.06, 95.51)	-37.8%	-3.9% (-5.14%, -2.64%)	85.3 (78.06, 95.51) → 54.29 (46.22, 65.34)	-36.4%	-4.02% (-4.57%, -3.47%)
		Male	914.7 (876.81, 957.27) → 198.34 (178.78, 224.06)	-78.3%	-9.21% (-10.46%, -7.93%)	198.34 (178.78, 224.06) → 132.82 (111.15, 159.69)	-33%	-3.61% (-4.14%, -3.07%)
HIV/AIDS - Drug-susceptible tuberculosis	Canada	Both	21.63 (13.45, 31.77) → 3.15 (1.97, 4.69)	-85.4%	-10.25% (-11.15%, -9.35%)	3.15 (1.97, 4.69) → 2.78 (1.74, 4.18)	-11.7%	-1.91% (-3.92%, 0.15%)
		Female	3.37 (2.1, 4.89) → 1.59 (0.99, 2.35)	-52.8%	-4.18% (-5.22%, -3.13%)	1.59 (0.99, 2.35) → 1.53 (0.93, 2.29)	-3.8%	-1.27% (-2.87%, 0.35%)
		Male	40.06 (24.94, 58.94) → 4.76 (2.96, 7.15)	-88.1%	-11.24% (-12.22%, -10.26%)	4.76 (2.96, 7.15) → 4.08 (2.54, 6.09)	-14.3%	-2.16% (-4.34%, 0.07%)
	France	Both	94.11 (64.42, 112.22) → 12.96 (8.22, 16.92)	-86.2%	-11.76% (-13.17%, -10.33%)	12.96 (8.22, 16.92) → 4.76 (2.93, 6.78)	-63.3%	-8.33% (-9.21%, -7.44%)
		Female	28.48 (20.05, 34.29) → 8.02 (5.03, 10.58)	-71.8%	-8.92% (-10.54%, -7.27%)	8.02 (5.03, 10.58) → 3.14 (1.99, 4.41)	-60.8%	-7.72% (-8.6%, -6.84%)
		Male	159.78 (108.8, 193.06) → 18.1 (11.52, 23.93)	-88.7%	-12.53% (-13.91%, -11.12%)	18.1 (11.52, 23.93) → 6.47 (3.93, 9.3)	-64.3%	-8.59% (-9.51%, -7.66%)
	Germany	Both	29.46 (18.82, 39.57) → 2.95 (1.82, 4.4)	-90%	-12.87% (-14.13%, -11.59%)	2.95 (1.82, 4.4) → 1.51 (0.91, 2.27)	-48.8%	-5.58% (-6.62%, -4.52%)
		Female	9.32 (6.13, 12.43) → 1.58 (0.98, 2.3)	-83%	-10.35% (-11.41%, -9.28%)	1.58 (0.98, 2.3) → 0.93 (0.56, 1.4)	-41.1%	-4.11% (-5.26%, -2.95%)
		Male	48.93 (31.13, 65.95) → 4.32 (2.66, 6.5)	-91.2%	-13.51% (-14.87%, -12.13%)	4.32 (2.66, 6.5) → 2.11 (1.26, 3.2)	-51.2%	-6.1% (-7.13%, -5.07%)

	Italy	Both	11.08 (6.78, 16.47) → 1.92 (1.16, 2.87)	-82.7%	-11.25% (-12.97%, -9.49%)	1.92 (1.16, 2.87) → 0.98 (0.59, 1.49)	-49%	-6.07% (-7.24%, -4.87%)
		Female	4.08 (2.56, 6.02) → 1 (0.61, 1.51)	-75.5%	-9.85% (-11.65%, -8.03%)	1 (0.61, 1.51) → 0.54 (0.34, 0.82)	-46%	-5.25% (-6.27%, -4.21%)
		Male	18.07 (11.04, 26.89) → 2.86 (1.72, 4.29)	-84.2%	-11.61% (-13.34%, -9.83%)	2.86 (1.72, 4.29) → 1.42 (0.85, 2.16)	-50.3%	-6.41% (-7.68%, -5.13%)
	Japan	Both	1.02 (0.9, 1.09) → 1.89 (1.2, 2.41)	85.3%	2.78% (1.48%, 4.1%)	1.89 (1.2, 2.41) → 1.47 (0.94, 1.96)	-22.2%	-1.9% (-2.76%, -1.02%)
		Female	0.59 (0.5, 0.65) → 0.96 (0.61, 1.24)	62.7%	2.1% (1.01%, 3.2%)	0.96 (0.61, 1.24) → 0.93 (0.58, 1.23)	-3.1%	0.31% (-0.72%, 1.36%)
		Male	1.46 (1.28, 1.56) → 2.82 (1.79, 3.6)	93.2%	3.01% (1.63%, 4.42%)	2.82 (1.79, 3.6) → 2.02 (1.29, 2.68)	-28.4%	-2.78% (-3.57%, -1.97%)
	United Kingdom	Both	10.93 (6.82, 15.4) → 4.05 (2.5, 6.11)	-62.9%	-5.98% (-6.86%, -5.08%)	4.05 (2.5, 6.11) → 1.87 (1.16, 2.86)	-53.8%	-6.01% (-7.33%, -4.67%)
		Female	2.23 (1.41, 3.04) → 3.02 (1.88, 4.53)	35.4%	0.9% (-0.43%, 2.26%)	3.02 (1.88, 4.53) → 1.44 (0.87, 2.19)	-52.3%	-5.43% (-6.89%, -3.94%)
		Male	19.71 (12.28, 27.77) → 5.1 (3.13, 7.75)	-74.1%	-8.02% (-9.04%, -6.99%)	5.1 (3.13, 7.75) → 2.33 (1.44, 3.56)	-54.3%	-6.3% (-7.71%, -4.87%)
	United States of America	Both	20.13 (12.66, 29.84) → 2.12 (1.35, 3.12)	-89.5%	-11.91% (-12.72%, -11.08%)	2.12 (1.35, 3.12) → 1.68 (1.06, 2.54)	-20.8%	-2.11% (-3.98%, -0.2%)
		Female	5.23 (3.29, 7.75) → 1.27 (0.81, 1.87)	-75.7%	-7.91% (-8.91%, -6.89%)	1.27 (0.81, 1.87) → 0.96 (0.6, 1.44)	-24.4%	-2.46% (-4.35%, -0.54%)
Male		35.32 (22.13, 52.47) → 3 (1.91, 4.42)	-91.5%	-12.96% (-13.85%, -12.06%)	3 (1.91, 4.42) → 2.41 (1.53, 3.69)	-19.7%	-2.01% (-3.87%, -0.1%)
HIV/AIDS - Extensively drug-resistant tuberculosis	Canada	Both	0 (0, 0) → 0.01 (0, 0.03)	Inf%	48.83% (12.29%, 97.24%)	0.01 (0, 0.03) → 0.02 (0.01, 0.06)	100%	2.57% (0.36%, 4.84%)
		Female	0 (0, 0) → 0.01 (0, 0.02)	Inf%	53.54% (18.56%, 98.82%)	0.01 (0, 0.02) → 0.01 (0, 0.03)	0%	3.11% (1.25%, 5%)
		Male	0 (0, 0) → 0.02 (0.01, 0.05)	Inf%	48.47% (11.04%, 98.53%)	0.02 (0.01, 0.05) → 0.03 (0.01, 0.09)	50%	2.35% (-0.02%, 4.79%)
	France	Both	0 (0, 0) → 0.08 (0.03, 0.19)	Inf%	57.7% (16.94%, 112.67%)	0.08 (0.03, 0.19) → 0.05 (0.02, 0.14)	-37.5%	-3.24% (-3.83%, -2.65%)
		Female	0 (0, 0) → 0.05 (0.02, 0.12)	Inf%	59.7% (20.01%, 112.52%)	0.05 (0.02, 0.12) → 0.04 (0.01, 0.1)	-20%	-2.63% (-3.29%, -1.97%)
		Male	0 (0, 0) → 0.11 (0.04, 0.26)	Inf%	57.44% (15.94%, 113.81%)	0.11 (0.04, 0.26) → 0.07 (0.02, 0.19)	-36.4%	-3.52% (-4.13%, -2.9%)
	Germany	Both	0 (0, 0) → 0.03 (0.01, 0.06)	Inf%	52.98% (15.66%, 102.34%)	0.03 (0.01, 0.06) → 0.03 (0.01, 0.1)	0%	2.62% (0.42%, 4.86%)
		Female	0 (0, 0) → 0.01 (0.01, 0.03)	Inf%	54.63% (18.42%, 101.92%)	0.01 (0.01, 0.03) → 0.02 (0, 0.06)	100%	4.22% (2.02%, 6.47%)
		Male	0 (0, 0) → 0.04 (0.01, 0.09)	Inf%	52.93% (14.87%, 103.58%)	0.04 (0.01, 0.09) → 0.05 (0.01, 0.14)	25%	2.04% (-0.17%, 4.29%)
	Italy	Both	0 (0, 0) → 0.03 (0.01, 0.06)	Inf%	56.9% (17.78%, 109.01%)	0.03 (0.01, 0.06) → 0.02 (0.01, 0.04)	-33.3%	-4.25% (-5.97%, -2.51%)
		Female	0 (0, 0) → 0.01 (0.01, 0.03)	Inf%	56.81% (18.99%, 106.65%)	0.01 (0.01, 0.03) → 0.01 (0, 0.02)	0%	-3.45% (-5.08%, -1.78%)
		Male	0 (0, 0) → 0.04 (0.02, 0.09)	Inf%	57.38% (17.43%, 110.92%)	0.04 (0.02, 0.09) → 0.02 (0.01, 0.06)	-50%	-4.6% (-6.36%, -2.8%)
	Japan	Both	0 (0, 0) → 0 (0, 0.02)	NaN%	54.42% (19.55%, 99.46%)	0 (0, 0.02) → 0.01 (0, 0.03)	Inf%	3.73% (3.12%, 4.33%)
		Female	0 (0, 0) → 0 (0, 0.01)	NaN%	52.67% (19.24%, 95.47%)	0 (0, 0.01) → 0 (0, 0.02)	NaN%	6.11% (5.33%, 6.89%)
		Male	0 (0, 0) → 0.01 (0, 0.02)	Inf%	55.35% (19.63%, 101.74%)	0.01 (0, 0.02) → 0.01 (0, 0.04)	0%	2.78% (2.22%, 3.34%)
	United Kingdom	Both	0 (0, 0) → 0.02 (0.01, 0.05)	Inf%	52.2% (16.39%, 99.03%)	0.02 (0.01, 0.05) → 0.02 (0, 0.04)	0%	-2.48% (-4.19%, -0.74%)
		Female	0 (0, 0) → 0.02 (0.01, 0.04)	Inf%	58.57% (23.33%, 103.87%)	0.02 (0.01, 0.04) → 0.01 (0, 0.03)	-50%	-1.87% (-3.49%, -0.22%)
		Male	0 (0, 0) → 0.03 (0.01, 0.06)	Inf%	50.18% (13.89%, 98.05%)	0.03 (0.01, 0.06) → 0.02 (0.01, 0.05)	-33.3%	-2.79% (-4.69%, -0.85%)

	United States of America	Both	0 (0, 0) → 0.01 (0, 0.02)	Inf%	40.43% (5.28%, 87.32%)	0.01 (0, 0.02) → 0.01 (0, 0.04)	0%	2.49% (0.39%, 4.63%)
		Female	0 (0, 0) → 0.01 (0, 0.01)	Inf%	43.11% (9.01%, 87.86%)	0.01 (0, 0.01) → 0.01 (0, 0.03)	0%	2.04% (-0.06%, 4.19%)
Male		0 (0, 0) → 0.02 (0.01, 0.03)	Inf%	39.95% (4.12%, 88.12%)	0.02 (0.01, 0.03) → 0.02 (0, 0.06)	0%	2.63% (0.53%, 4.77%)
HIV/AIDS - Multidrug-resistant tuberculosis without extensive drug resistance	Canada	Both	0.51 (0.1, 1.47) → 0.1 (0.04, 0.23)	-80.4%	-9.36% (-10.66%, -8.05%)	0.1 (0.04, 0.23) → 0.09 (0.03, 0.25)	-10%	-2.14% (-4.38%, 0.15%)
		Female	0.09 (0.02, 0.25) → 0.05 (0.02, 0.12)	-44.4%	-3.58% (-5.11%, -2.03%)	0.05 (0.02, 0.12) → 0.05 (0.02, 0.14)	0%	-1.62% (-3.5%, 0.29%)
		Male	0.93 (0.19, 2.71) → 0.15 (0.06, 0.34)	-83.9%	-10.37% (-11.73%, -9%)	0.15 (0.06, 0.34) → 0.14 (0.04, 0.35)	-6.7%	-2.35% (-4.75%, 0.11%)
	France	Both	1.21 (0.17, 4.19) → 0.58 (0.22, 1.28)	-52.1%	-5.83% (-7.52%, -4.12%)	0.58 (0.22, 1.28) → 0.23 (0.08, 0.59)	-60.3%	-7.64% (-8.32%, -6.96%)
		Female	0.38 (0.05, 1.25) → 0.37 (0.13, 0.81)	-2.6%	-2.89% (-4.89%, -0.84%)	0.37 (0.13, 0.81) → 0.15 (0.05, 0.41)	-59.5%	-7.06% (-7.75%, -6.37%)
		Male	2.04 (0.29, 7.16) → 0.8 (0.3, 1.76)	-60.8%	-6.66% (-8.31%, -4.99%)	0.8 (0.3, 1.76) → 0.31 (0.11, 0.79)	-61.3%	-7.9% (-8.62%, -7.18%)
	Germany	Both	0.53 (0.07, 1.97) → 0.19 (0.08, 0.41)	-64.2%	-6.92% (-8.16%, -5.66%)	0.19 (0.08, 0.41) → 0.15 (0.03, 0.39)	-21.1%	-2.09% (-4.02%, -0.12%)
		Female	0.17 (0.02, 0.61) → 0.1 (0.04, 0.22)	-41.2%	-4.29% (-5.64%, -2.91%)	0.1 (0.04, 0.22) → 0.09 (0.02, 0.25)	-10%	-0.56% (-2.51%, 1.43%)
		Male	0.87 (0.12, 3.3) → 0.28 (0.11, 0.6)	-67.8%	-7.6% (-8.89%, -6.3%)	0.28 (0.11, 0.6) → 0.2 (0.04, 0.54)	-28.6%	-2.64% (-4.58%, -0.66%)
	Italy	Both	0.28 (0.03, 1) → 0.2 (0.08, 0.42)	-28.6%	-3.47% (-6.2%, -0.67%)	0.2 (0.08, 0.42) → 0.07 (0.02, 0.17)	-65%	-8.63% (-10.38%, -6.85%)
		Female	0.1 (0.01, 0.37) → 0.1 (0.04, 0.22)	0%	-2.02% (-5.16%, 1.23%)	0.1 (0.04, 0.22) → 0.04 (0.01, 0.1)	-60%	-7.86% (-9.51%, -6.19%)
		Male	0.45 (0.05, 1.64) → 0.29 (0.12, 0.62)	-35.6%	-3.85% (-6.47%, -1.16%)	0.29 (0.12, 0.62) → 0.1 (0.03, 0.25)	-65.5%	-8.96% (-10.76%, -7.11%)
	Japan	Both	0.01 (0, 0.05) → 0.03 (0.01, 0.1)	200%	0.94% (-3.6%, 5.71%)	0.03 (0.01, 0.1) → 0.03 (0.01, 0.12)	0%	-0.94% (-1.54%, -0.33%)
		Female	0.01 (0, 0.03) → 0.02 (0.01, 0.05)	100%	0.24% (-4.08%, 4.75%)	0.02 (0.01, 0.05) → 0.02 (0, 0.07)	0%	1.32% (0.55%, 2.1%)
		Male	0.02 (0, 0.07) → 0.05 (0.01, 0.15)	150%	1.19% (-3.45%, 6.04%)	0.05 (0.01, 0.15) → 0.04 (0.01, 0.16)	-20%	-1.84% (-2.39%, -1.29%)
	United Kingdom	Both	0.28 (0.06, 0.81) → 0.15 (0.06, 0.31)	-46.4%	-6.04% (-7.79%, -4.26%)	0.15 (0.06, 0.31) → 0.06 (0.02, 0.17)	-60%	-6.92% (-8.39%, -5.42%)
		Female	0.06 (0.01, 0.17) → 0.11 (0.04, 0.23)	83.3%	0.79% (-0.92%, 2.52%)	0.11 (0.04, 0.23) → 0.05 (0.02, 0.13)	-54.5%	-6.34% (-7.78%, -4.89%)
		Male	0.51 (0.11, 1.46) → 0.18 (0.07, 0.39)	-64.7%	-8.12% (-10%, -6.2%)	0.18 (0.07, 0.39) → 0.08 (0.03, 0.21)	-55.6%	-7.21% (-8.86%, -5.53%)
	United States of America	Both	1.71 (0.56, 4.2) → 0.08 (0.03, 0.16)	-95.3%	-16.46% (-18.14%, -14.75%)	0.08 (0.03, 0.16) → 0.06 (0.01, 0.18)	-25%	-2.2% (-4.37%, 0.01%)
		Female	0.46 (0.15, 1.14) → 0.05 (0.02, 0.09)	-89.1%	-12.81% (-13.95%, -11.65%)	0.05 (0.02, 0.09) → 0.04 (0.01, 0.11)	-20%	-2.62% (-4.79%, -0.4%)
		Male	2.98 (0.99, 7.3) → 0.11 (0.04, 0.22)	-96.3%	-17.46% (-19.27%, -15.61%)	0.11 (0.04, 0.22) → 0.09 (0.02, 0.25)	-18.2%	-2.08% (-4.24%, 0.13%)
HIV/AIDS resulting in other diseases	Canada	Both	119.31 (103.23, 135.88) → 46.27 (38.22, 58.37)	-61.2%	-6.74% (-8.16%, -5.3%)	46.27 (38.22, 58.37) → 28.46 (22.48, 38.19)	-38.5%	-4.31% (-5.16%, -3.46%)
		Female	18.74 (15.8, 21.5) → 23.08 (18.11, 29.69)	23.2%	-0.53% (-1.77%, 0.73%)	23.08 (18.11, 29.69) → 16.11 (11.53, 23.02)	-30.2%	-3.29% (-3.77%, -2.81%)
		Male	220.77 (188.88, 252.02) → 70.3 (57.47, 89.63)	-68.2%	-7.75% (-9.24%, -6.23%)	70.3 (57.47, 89.63) → 41.26 (32.42, 54.55)	-41.3%	-4.7% (-5.68%, -3.71%)
	France	Both	154.58 (131.04, 191.4) → 47.86 (39.89, 57.28)	-69%	-8.56% (-10.4%, -6.68%)	47.86 (39.89, 57.28) → 37.54 (30.69, 46.73)	-21.6%	-2.32% (-2.62%, -2.02%)
		Female	47.16 (39.98, 59.59) → 25.09 (20.19, 30.71)	-46.8%	-6.38% (-8.26%, -4.45%)	25.09 (20.19, 30.71) → 20.16 (15.5, 25.07)	-19.6%	-2.35% (-2.72%, -1.98%)
		Male	262.22 (219.3, 326.74) → 71.66 (58.48, 86.21)	-72.7%	-9.07% (-10.92%, -7.17%)	71.66 (58.48, 86.21) → 55.92 (44.45, 69.79)	-22%	-2.27% (-2.56%, -1.98%)
	Germany	Both	64.39 (52.35, 78.81) → 28.74 (25.05, 32.49)	-55.4%	-6.98% (-8.74%, -5.18%)	28.74 (25.05, 32.49) → 17.69 (14.67, 21.15)	-38.4%	-3.81% (-4.73%, -2.88%)
		Female	20.72 (16.67, 25.8) → 15.54 (13.25, 18.36)	-25%	-4.34% (-5.9%, -2.77%)	15.54 (13.25, 18.36) → 11.44 (8.73, 14.38)	-26.4%	-2.01% (-3.03%, -0.98%)
		Male	106.61 (85.33, 132.89) → 41.87 (35.81, 48.35)	-60.7%	-7.65% (-9.49%, -5.76%)	41.87 (35.81, 48.35) → 24.04 (19.98, 29.04)	-42.6%	-4.51% (-5.41%, -3.59%)
	Italy	Both	167.77 (155.94, 180.77) → 69.68 (63.02, 79.86)	-58.5%	-7.8% (-9.74%, -5.83%)	69.68 (63.02, 79.86) → 39.51 (34.53, 46.37)	-43.3%	-5.21% (-5.48%, -4.94%)
		Female	61.62 (55.12, 67.54) → 36.69 (32.46, 42.35)	-40.5%	-6.36% (-8.39%, -4.28%)	36.69 (32.46, 42.35) → 23 (19.84, 27.5)	-37.3%	-4.08% (-4.53%, -3.64%)
		Male	273.75 (250.76, 297.84) → 103.71 (93.19, 120.9)	-62.1%	-8.17% (-10.1%, -6.2%)	103.71 (93.19, 120.9) → 56.48 (49.62, 66.72)	-45.5%	-5.67% (-5.99%, -5.34%)
	Japan	Both	1.48 (1.34, 1.68) → 5.04 (4.21, 6.06)	240.5%	5.94% (4.92%, 6.97%)	5.04 (4.21, 6.06) → 5.29 (4.28, 6.6)	5%	0.64% (0.28%, 1%)
		Female	0.85 (0.77, 0.96) → 2.7 (2.21, 3.28)	217.6%	5.59% (4.77%, 6.41%)	2.7 (2.21, 3.28) → 3.34 (2.71, 4.12)	23.7%	2.32% (1.89%, 2.76%)
		Male	2.11 (1.9, 2.42) → 7.37 (6.15, 8.8)	249.3%	6.06% (4.96%, 7.17%)	7.37 (6.15, 8.8) → 7.24 (5.87, 9.02)	-1.8%	-0.05% (-0.4%, 0.31%)
	United Kingdom	Both	33.94 (28.81, 38.79) → 27.87 (24.46, 32.49)	-17.9%	-3.19% (-5%, -1.35%)	27.87 (24.46, 32.49) → 23.5 (19.56, 28.54)	-15.7%	-1.26% (-1.8%, -0.73%)
		Female	6.79 (5.73, 7.72) → 20.14 (17.31, 23.62)	196.6%	3.85% (2.29%, 5.44%)	20.14 (17.31, 23.62) → 17.11 (14.25, 20.59)	-15%	-0.94% (-1.87%, 0%)
		Male	61.32 (52.11, 70.58) → 35.84 (31.22, 41.26)	-41.6%	-5.2% (-7.18%, -3.18%)	35.84 (31.22, 41.26) → 30.23 (24.84, 37.02)	-15.7%	-1.39% (-1.81%, -0.97%)
	United States of America	Both	500.3 (477.66, 524.32) → 138.72 (125.89, 154.53)	-72.3%	-7.97% (-9.15%, -6.78%)	138.72 (125.89, 154.53) → 91.44 (77.58, 108.41)	-34.1%	-3.73% (-4.25%, -3.21%)
		Female	131.34 (125.42, 137.54) → 83.98 (76.94, 94.2)	-36.1%	-3.79% (-5.05%, -2.51%)	83.98 (76.94, 94.2) → 53.29 (45.15, 64.22)	-36.5%	-4.04% (-4.57%, -3.51%)
		Male	876.4 (835.86, 920.91) → 195.22 (175.99, 220.81)	-77.7%	-9.1% (-10.37%, -7.81%)	195.22 (175.99, 220.81) → 130.3 (108.59, 157.12)	-33.3%	-3.63% (-4.15%, -3.12%)

**Table 5:** Results of DALYs number in G7 countries.

Cause	Country	Sex	Value_ (1990-2010)	PC	AAPC_UI_ (1990-2010)	Value_ (2010-2021)	PC	AAPC_UI_ (2010-2021)
HIV/AIDS	Canada	Both	103.11 (62.96, 145.08) → 177.51 (107.15, 271.55)	72.2%	1.55% (0.77%, 2.34%)	177.51 (107.15, 271.55) → 215.54 (125.27, 337.64)	21.4%	1.99% (1.63%, 2.34%)
		Female	11.35 (5.42, 17.29) → 44.28 (21.07, 76.49)	290.1%	6.08% (5.06%, 7.12%)	44.28 (21.07, 76.49) → 51.89 (17.94, 99.62)	17.2%	1.56% (1.14%, 1.99%)
		Male	91.76 (49.94, 132.26) → 133.23 (74.1, 205.78)	45.2%	0.57% (-0.42%, 1.57%)	133.23 (74.1, 205.78) → 163.65 (93.81, 263.96)	22.8%	2.13% (1.74%, 2.51%)
	France	Both	420.98 (247.16, 645.92) → 913.11 (578.91, 1404.02)	116.9%	3.06% (2.58%, 3.54%)	913.11 (578.91, 1404.02) → 1254.35 (815.52, 1857.97)	37.4%	3.06% (2.8%, 3.31%)
		Female	80.61 (38, 127.59) → 179.98 (91.14, 289.69)	123.3%	2.86% (2.14%, 3.58%)	179.98 (91.14, 289.69) → 297.17 (153.31, 479.01)	65.1%	4.35% (4.05%, 4.66%)
		Male	340.37 (175.77, 550.52) → 733.13 (428.29, 1182)	115.4%	3.11% (2.66%, 3.58%)	733.13 (428.29, 1182) → 957.18 (575.86, 1468.09)	30.6%	2.71% (2.37%, 3.05%)
	Germany	Both	203.41 (128.58, 282.78) → 379.63 (255.93, 556.06)	86.6%	1.85% (0.55%, 3.17%)	379.63 (255.93, 556.06) → 540.89 (339.08, 784.27)	42.5%	3.73% (2.17%, 5.32%)
		Female	52.55 (24.29, 83.23) → 112.03 (59.14, 188.09)	113.2%	2.4% (1.58%, 3.23%)	112.03 (59.14, 188.09) → 135.36 (53.15, 260.52)	20.8%	1.93% (1.06%, 2.81%)
		Male	150.86 (86.94, 222.55) → 267.61 (162.04, 393.63)	77.4%	1.64% (0.02%, 3.28%)	267.61 (162.04, 393.63) → 405.52 (241.49, 612.52)	51.5%	4.38% (2.55%, 6.25%)
	Italy	Both	210.9 (155.01, 288.65) → 586.55 (440.36, 804.87)	178.1%	3.73% (3.06%, 4.41%)	586.55 (440.36, 804.87) → 724.37 (520.79, 1010.63)	23.5%	2.52% (0.94%, 4.13%)
		Female	47.82 (33.18, 64.99) → 156.94 (95.59, 266.68)	228.2%	4.32% (3.46%, 5.19%)	156.94 (95.59, 266.68) → 195.28 (103.58, 332.88)	24.4%	2.62% (0.88%, 4.39%)
		Male	163.08 (115.11, 232.42) → 429.61 (320, 599.64)	163.4%	3.54% (2.91%, 4.18%)	429.61 (320, 599.64) → 529.09 (377.29, 733.67)	23.2%	2.49% (0.96%, 4.04%)
	Japan	Both	11.39 (9.45, 13.94) → 135.12 (93.7, 187.99)	1086.3%	13.26% (13.04%, 13.47%)	135.12 (93.7, 187.99) → 427.47 (254.98, 680.73)	216.4%	11.32% (10.99%, 11.64%)
		Female	3.83 (2.97, 4.82) → 40.42 (27.06, 60.51)	955.4%	12.16% (11.98%, 12.34%)	40.42 (27.06, 60.51) → 139.38 (80.97, 221.17)	244.8%	12.11% (11.74%, 12.49%)
		Male	7.56 (6.22, 9.29) → 94.7 (67.48, 133.12)	1152.6%	13.76% (13.46%, 14.07%)	94.7 (67.48, 133.12) → 288.09 (171.64, 447.96)	204.2%	10.95% (10.64%, 11.27%)
	United Kingdom	Both	92.04 (76.94, 110.8) → 193.26 (140.93, 264.85)	110%	2.16% (0.83%, 3.5%)	193.26 (140.93, 264.85) → 464.43 (306.59, 657.98)	140.3%	8.26% (7.97%, 8.55%)
		Female	9.16 (7.12, 11.77) → 50.11 (33.02, 72.4)	447.1%	7.63% (6.74%, 8.54%)	50.11 (33.02, 72.4) → 140.18 (82.96, 214.2)	179.7%	9.94% (9.58%, 10.3%)
		Male	82.88 (69.33, 99.1) → 143.15 (104.8, 192.85)	72.7%	0.95% (-0.58%, 2.51%)	143.15 (104.8, 192.85) → 324.25 (213.82, 453.6)	126.5%	7.62% (7.31%, 7.93%)
	United States of America	Both	1552.82 (1327.34, 1813.59) → 3333.88 (2612.32, 4282.74)	114.7%	2.68% (2.09%, 3.27%)	3333.88 (2612.32, 4282.74) → 7016.64 (4995.08, 9770.23)	110.5%	7.07% (6.84%, 7.3%)
		Female	467.81 (386.25, 550.28) → 852.68 (640.51, 1128.87)	82.3%	1.74% (1.03%, 2.47%)	852.68 (640.51, 1128.87) → 1439.64 (926.71, 2220.31)	68.8%	4.82% (4.41%, 5.22%)
		Male	1085.01 (911.95, 1283.44) → 2481.2 (1932.95, 3200.7)	128.7%	3.07% (2.46%, 3.68%)	2481.2 (1932.95, 3200.7) → 5577 (3954.31, 7741.59)	124.8%	7.74% (7.54%, 7.94%)
HIV/AIDS - Drug-susceptible tuberculosis	Canada	Both	14.79 (9.12, 21.47) → 7.07 (4.48, 10.29)	-52.2%	-4.27% (-4.69%, -3.84%)	7.07 (4.48, 10.29) → 8.64 (5.33, 12.65)	22.2%	1.58% (0.28%, 2.89%)
		Female	1.63 (1.02, 2.34) → 1.37 (0.89, 1.92)	-16%	-1.15% (-2.57%, 0.3%)	1.37 (0.89, 1.92) → 1.75 (1.1, 2.53)	27.7%	1.93% (0.78%, 3.08%)
		Male	13.16 (8.07, 19.18) → 5.7 (3.59, 8.35)	-56.7%	-4.86% (-5.34%, -4.38%)	5.7 (3.59, 8.35) → 6.89 (4.08, 10.15)	20.9%	1.49% (0.16%, 2.84%)
	France	Both	99.29 (69.74, 125.44) → 73.48 (47.48, 101.6)	-26%	-2.25% (-2.92%, -1.59%)	73.48 (47.48, 101.6) → 41.6 (23.95, 60.39)	-43.4%	-4.57% (-5.42%, -3.71%)
		Female	18.78 (10.47, 26.11) → 17.2 (8.93, 25.17)	-8.4%	-1.04% (-1.97%, -0.1%)	17.2 (8.93, 25.17) → 9.43 (3.42, 14.13)	-45.2%	-4.71% (-5.58%, -3.83%)
		Male	80.51 (54.88, 104.17) → 56.29 (35.62, 80.51)	-30.1%	-2.59% (-3.22%, -1.96%)	56.29 (35.62, 80.51) → 32.17 (14.73, 47.87)	-42.8%	-4.53% (-5.39%, -3.66%)
	Germany	Both	54.96 (36.08, 75.39) → 21.4 (13.83, 30.04)	-61.1%	-5.36% (-6.16%, -4.55%)	21.4 (13.83, 30.04) → 19.59 (12.79, 27.37)	-8.5%	-0.01% (-2.13%, 2.16%)
		Female	13.31 (6.73, 18.88) → 5.2 (3.2, 7.27)	-60.9%	-5.53% (-6.21%, -4.84%)	5.2 (3.2, 7.27) → 3.86 (2.23, 5.4)	-25.8%	-2.56% (-3.33%, -1.79%)
		Male	41.64 (25.71, 59.15) → 16.2 (10.43, 22.91)	-61.1%	-5.28% (-6.35%, -4.2%)	16.2 (10.43, 22.91) → 15.74 (10.19, 22)	-2.8%	0.66% (-1.82%, 3.21%)

	Italy	Both	9.51 (6.21, 13.08) → 9.51 (6.07, 13.42)	0%	-1.04% (-1.79%, -0.29%)	9.51 (6.07, 13.42) → 8.03 (5.22, 11.73)	-15.6%	-0.93% (-2.06%, 0.21%)
		Female	2.27 (1.43, 3.25) → 2.34 (1.47, 3.3)	3.1%	-0.9% (-1.79%, 0.01%)	2.34 (1.47, 3.3) → 1.89 (1.2, 2.76)	-19.2%	-1.49% (-2.49%, -0.48%)
		Male	7.24 (4.75, 10.01) → 7.17 (4.56, 10.18)	-1%	-1.09% (-1.86%, -0.31%)	7.17 (4.56, 10.18) → 6.14 (3.97, 9.02)	-14.4%	-0.76% (-1.94%, 0.43%)
	Japan	Both	4.22 (3.51, 4.85) → 20.08 (13.86, 25.89)	375.8%	8.32% (7.74%, 8.91%)	20.08 (13.86, 25.89) → 27.75 (18.48, 36.61)	38.2%	3.55% (2.78%, 4.32%)
		Female	1.37 (1.07, 1.65) → 5.12 (3.33, 6.79)	273.7%	6.66% (5.96%, 7.36%)	5.12 (3.33, 6.79) → 8.51 (5.37, 11.58)	66.2%	5.5% (4.47%, 6.55%)
		Male	2.85 (2.34, 3.31) → 14.96 (10.29, 19.28)	424.9%	9.01% (8.44%, 9.58%)	14.96 (10.29, 19.28) → 19.24 (12.94, 25.26)	28.6%	2.8% (2.08%, 3.53%)
	United Kingdom	Both	18.51 (12.07, 25.23) → 9.81 (6.39, 13.64)	-47%	-4.28% (-5%, -3.55%)	9.81 (6.39, 13.64) → 7.93 (5.4, 10.69)	-19.2%	-1.46% (-2.66%, -0.24%)
		Female	1.64 (1.12, 2.2) → 2.45 (1.61, 3.4)	49.4%	1.41% (0.29%, 2.53%)	2.45 (1.61, 3.4) → 2.14 (1.46, 2.84)	-12.7%	-0.35% (-1.49%, 0.81%)
		Male	16.86 (10.93, 23.16) → 7.36 (4.78, 10.25)	-56.3%	-5.46% (-6.31%, -4.6%)	7.36 (4.78, 10.25) → 5.79 (3.95, 7.78)	-21.3%	-1.82% (-3.17%, -0.45%)
	United States of America	Both	53.26 (33.63, 77.13) → 37.78 (24.29, 53.84)	-29.1%	-2.37% (-2.78%, -1.96%)	37.78 (24.29, 53.84) → 81.56 (52.89, 118.58)	115.9%	7.69% (6.32%, 9.09%)
		Female	15.75 (9.95, 23.15) → 8.98 (5.69, 12.79)	-43%	-3.48% (-4.21%, -2.74%)	8.98 (5.69, 12.79) → 14.4 (9.49, 20.66)	60.4%	4.69% (3.13%, 6.28%)
Male		37.51 (23.85, 54.58) → 28.8 (18.53, 41.02)	-23.2%	-1.94% (-2.26%, -1.62%)	28.8 (18.53, 41.02) → 67.17 (43.52, 97.97)	133.2%	8.47% (7.17%, 9.79%)
HIV/AIDS - Extensively drug-resistant tuberculosis	Canada	Both	0 (0, 0) → 0.03 (0.01, 0.07)	Inf%	57.65% (19.6%, 107.81%)	0.03 (0.01, 0.07) → 0.06 (0.02, 0.17)	100%	5.92% (4.46%, 7.41%)
		Female	0 (0, 0) → 0.01 (0, 0.01)	Inf%	56.62% (21.81%, 101.36%)	0.01 (0, 0.01) → 0.01 (0, 0.03)	0%	6.61% (5.29%, 7.95%)
		Male	0 (0, 0) → 0.03 (0.01, 0.06)	Inf%	56.74% (19.22%, 106.07%)	0.03 (0.01, 0.06) → 0.05 (0.01, 0.14)	66.7%	5.76% (4.25%, 7.29%)
	France	Both	0 (0, 0) → 0.42 (0.15, 0.97)	Inf%	63.55% (22.47%, 118.41%)	0.42 (0.15, 0.97) → 0.42 (0.14, 1.06)	0%	0.53% (-0.24%, 1.31%)
		Female	0 (0, 0) → 0.1 (0.03, 0.23)	Inf%	62.56% (24.44%, 112.36%)	0.1 (0.03, 0.23) → 0.09 (0.02, 0.24)	-10%	0.1% (-0.74%, 0.94%)
		Male	0 (0, 0) → 0.32 (0.11, 0.74)	Inf%	62.43% (22.13%, 116.02%)	0.32 (0.11, 0.74) → 0.33 (0.08, 0.83)	3.1%	0.66% (-0.12%, 1.45%)
	Germany	Both	0 (0, 0) → 0.17 (0.07, 0.39)	Inf%	62.17% (22.29%, 115.05%)	0.17 (0.07, 0.39) → 0.36 (0.07, 1.08)	111.8%	8.11% (4.03%, 12.34%)
		Female	0 (0, 0) → 0.04 (0.01, 0.09)	Inf%	57.55% (20.65%, 105.74%)	0.04 (0.01, 0.09) → 0.07 (0.01, 0.21)	75%	5.77% (3.57%, 8.01%)
		Male	0 (0, 0) → 0.13 (0.05, 0.3)	Inf%	62.23% (23.1%, 113.8%)	0.13 (0.05, 0.3) → 0.29 (0.06, 0.86)	123.1%	8.66% (4.15%, 13.38%)
	Italy	Both	0 (0, 0) → 0.13 (0.05, 0.28)	Inf%	67.04% (27.09%, 119.53%)	0.13 (0.05, 0.28) → 0.12 (0.04, 0.29)	-7.7%	0.83% (-0.98%, 2.67%)
		Female	0 (0, 0) → 0.03 (0.01, 0.06)	Inf%	65.19% (27.8%, 113.51%)	0.03 (0.01, 0.06) → 0.03 (0.01, 0.07)	0%	0.2% (-1.56%, 1.99%)
		Male	0 (0, 0) → 0.1 (0.04, 0.21)	Inf%	66.18% (27.13%, 117.23%)	0.1 (0.04, 0.21) → 0.09 (0.03, 0.22)	-10%	1.02% (-0.82%, 2.89%)
	Japan	Both	0 (0, 0) → 0.05 (0.01, 0.14)	Inf%	63.2% (24.91%, 113.25%)	0.05 (0.01, 0.14) → 0.11 (0.02, 0.48)	120%	8.88% (7.73%, 10.03%)
		Female	0 (0, 0) → 0.01 (0, 0.04)	Inf%	57.21% (22.24%, 102.18%)	0.01 (0, 0.04) → 0.03 (0.01, 0.15)	200%	11.36% (10.25%, 12.49%)
		Male	0 (0, 0) → 0.03 (0.01, 0.11)	Inf%	64.04% (26.23%, 113.16%)	0.03 (0.01, 0.11) → 0.08 (0.02, 0.33)	166.7%	7.95% (6.7%, 9.22%)

	United Kingdom	Both	0 (0, 0) → 0.04 (0.02, 0.1)	Inf%	50.75% (14.69%, 98.15%)	0.04 (0.02, 0.1) → 0.05 (0.02, 0.12)	25%	0.69% (-1.37%, 2.79%)
		Female	0 (0, 0) → 0.01 (0, 0.02)	Inf%	52.75% (19.91%, 94.59%)	0.01 (0, 0.02) → 0.01 (0, 0.03)	0%	1.25% (-0.47%, 2.99%)
		Male	0 (0, 0) → 0.03 (0.01, 0.07)	Inf%	49.03% (13.58%, 95.55%)	0.03 (0.01, 0.07) → 0.03 (0.01, 0.09)	0%	0.52% (-1.74%, 2.84%)
	United States of America	Both	0 (0, 0) → 0.18 (0.07, 0.4)	Inf%	52.83% (13.31%, 106.14%)	0.18 (0.07, 0.4) → 0.67 (0.13, 2.05)	272.2%	12.77% (11.23%, 14.33%)
		Female	0 (0, 0) → 0.04 (0.02, 0.1)	Inf%	48.4% (11.76%, 97.05%)	0.04 (0.02, 0.1) → 0.11 (0.02, 0.35)	175%	9.28% (7.52%, 11.07%)
Male		0 (0, 0) → 0.14 (0.05, 0.3)	Inf%	52.97% (14.13%, 105.03%)	0.14 (0.05, 0.3) → 0.56 (0.11, 1.69)	300%	13.65% (12.19%, 15.12%)
HIV/AIDS - Multidrug-resistant tuberculosis without extensive drug resistance	Canada	Both	0.38 (0.08, 1.09) → 0.23 (0.09, 0.5)	-39.5%	-4.04% (-4.97%, -3.1%)	0.23 (0.09, 0.5) → 0.28 (0.09, 0.73)	21.7%	1.12% (-0.41%, 2.67%)
		Female	0.04 (0.01, 0.12) → 0.04 (0.02, 0.09)	0%	-1.09% (-2.96%, 0.81%)	0.04 (0.02, 0.09) → 0.06 (0.02, 0.14)	50%	1.68% (0.31%, 3.07%)
		Male	0.34 (0.07, 0.96) → 0.19 (0.07, 0.41)	-44.1%	-4.58% (-5.47%, -3.69%)	0.19 (0.07, 0.41) → 0.22 (0.07, 0.59)	15.8%	0.99% (-0.58%, 2.58%)
	France	Both	1.01 (0.15, 3.46) → 3.08 (1.2, 6.38)	205%	5.17% (3.82%, 6.55%)	3.08 (1.2, 6.38) → 1.83 (0.65, 4.63)	-40.6%	-3.99% (-4.72%, -3.26%)
		Female	0.2 (0.03, 0.72) → 0.73 (0.24, 1.54)	265%	6.18% (4.37%, 8.01%)	0.73 (0.24, 1.54) → 0.41 (0.1, 1.05)	-43.8%	-4.31% (-5.1%, -3.52%)
		Male	0.81 (0.12, 2.81) → 2.36 (0.89, 5.09)	191.4%	4.88% (3.64%, 6.13%)	2.36 (0.89, 5.09) → 1.43 (0.4, 3.71)	-39.4%	-3.9% (-4.63%, -3.16%)
	Germany	Both	0.96 (0.13, 3.44) → 1.29 (0.55, 2.6)	34.4%	0.66% (-0.03%, 1.35%)	1.29 (0.55, 2.6) → 1.65 (0.35, 4.59)	27.9%	3.38% (-0.23%, 7.12%)
		Female	0.23 (0.03, 0.83) → 0.3 (0.13, 0.59)	30.4%	0.32% (-0.89%, 1.55%)	0.3 (0.13, 0.59) → 0.32 (0.06, 0.9)	6.7%	1.03% (-0.9%, 3.01%)
		Male	0.74 (0.1, 2.63) → 0.99 (0.42, 2.01)	33.8%	0.79% (-0.01%, 1.59%)	0.99 (0.42, 2.01) → 1.33 (0.28, 3.62)	34.3%	3.96% (-0.06%, 8.13%)
	Italy	Both	0.22 (0.02, 0.81) → 0.92 (0.41, 1.85)	318.2%	7.82% (4.97%, 10.76%)	0.92 (0.41, 1.85) → 0.51 (0.2, 1.27)	-44.6%	-3.78% (-5.45%, -2.07%)
		Female	0.05 (0.01, 0.2) → 0.22 (0.1, 0.45)	340%	7.63% (4.27%, 11.1%)	0.22 (0.1, 0.45) → 0.12 (0.05, 0.29)	-45.5%	-4.37% (-6.01%, -2.7%)
		Male	0.16 (0.02, 0.62) → 0.7 (0.31, 1.41)	337.5%	7.89% (5.22%, 10.64%)	0.7 (0.31, 1.41) → 0.4 (0.15, 0.98)	-42.9%	-3.6% (-5.29%, -1.87%)
	Japan	Both	0.05 (0.01, 0.19) → 0.34 (0.11, 0.98)	580%	6.15% (2.16%, 10.31%)	0.34 (0.11, 0.98) → 0.5 (0.12, 1.97)	47.1%	4.19% (3.28%, 5.11%)
		Female	0.02 (0, 0.06) → 0.08 (0.03, 0.25)	300%	4.55% (0.44%, 8.83%)	0.08 (0.03, 0.25) → 0.15 (0.04, 0.6)	87.5%	6.44% (5.45%, 7.43%)
		Male	0.04 (0.01, 0.13) → 0.25 (0.08, 0.73)	525%	6.79% (2.83%, 10.9%)	0.25 (0.08, 0.73) → 0.34 (0.08, 1.38)	36%	3.35% (2.36%, 4.34%)
	United Kingdom	Both	0.46 (0.1, 1.33) → 0.32 (0.13, 0.65)	-30.4%	-4.71% (-6.33%, -3.07%)	0.32 (0.13, 0.65) → 0.22 (0.09, 0.55)	-31.2%	-3.34% (-5.02%, -1.63%)
		Female	0.04 (0.01, 0.11) → 0.08 (0.03, 0.16)	100%	1.29% (-0.26%, 2.88%)	0.08 (0.03, 0.16) → 0.06 (0.02, 0.14)	-25%	-2.56% (-3.96%, -1.15%)
		Male	0.42 (0.1, 1.22) → 0.24 (0.1, 0.49)	-42.9%	-5.91% (-7.66%, -4.14%)	0.24 (0.1, 0.49) → 0.16 (0.06, 0.41)	-33.3%	-3.58% (-5.45%, -1.67%)
	United States of America	Both	4.77 (1.66, 11.35) → 1.31 (0.56, 2.68)	-72.5%	-7.85% (-9.19%, -6.49%)	1.31 (0.56, 2.68) → 2.89 (0.63, 8.49)	120.6%	7.58% (5.93%, 9.26%)
		Female	1.41 (0.49, 3.29) → 0.31 (0.14, 0.63)	-78%	-8.86% (-9.88%, -7.83%)	0.31 (0.14, 0.63) → 0.5 (0.11, 1.5)	61.3%	4.36% (2.5%, 6.25%)
		Male	3.36 (1.17, 8.01) → 1 (0.42, 2.05)	-70.2%	-7.46% (-8.93%, -5.96%)	1 (0.42, 2.05) → 2.39 (0.51, 7.01)	139%	8.4% (6.83%, 10%)
HIV/AIDS resulting in other diseases	Canada	Both	87.94 (46.08, 130.75) → 170.18 (99.66, 264.14)	93.5%	2.07% (1.24%, 2.91%)	170.18 (99.66, 264.14) → 206.56 (116.82, 328.41)	21.4%	2% (1.64%, 2.36%)
		Female	9.68 (3.93, 15.9) → 42.86 (19.88, 75.1)	342.8%	6.67% (5.61%, 7.75%)	42.86 (19.88, 75.1) → 50.08 (16.34, 97.8)	16.8%	1.55% (1.11%, 1.99%)
		Male	78.26 (35.2, 119.21) → 127.32 (68.15, 198.79)	62.7%	1.07% (0.03%, 2.12%)	127.32 (68.15, 198.79) → 156.49 (86.63, 256.67)	22.9%	2.15% (1.77%, 2.54%)
	France	Both	320.68 (157.88, 537.8) → 836.12 (504.27, 1321.61)	160.7%	4.01% (3.54%, 4.48%)	836.12 (504.27, 1321.61) → 1210.5 (773.53, 1806.97)	44.8%	3.51% (3.24%, 3.79%)
		Female	61.63 (22.79, 108.12) → 161.96 (75.33, 271.66)	162.8%	3.6% (2.87%, 4.33%)	161.96 (75.33, 271.66) → 287.24 (144.88, 466.92)	77.4%	4.95% (4.65%, 5.26%)
		Male	259.05 (107.1, 451.31) → 674.16 (374.35, 1124.6)	160.2%	4.12% (3.68%, 4.55%)	674.16 (374.35, 1124.6) → 923.26 (541.57, 1433.37)	36.9%	3.13% (2.76%, 3.5%)
	Germany	Both	147.5 (86.11, 227.77) → 356.77 (229.58, 534.29)	141.9%	2.96% (1.63%, 4.32%)	356.77 (229.58, 534.29) → 519.29 (317.32, 763.97)	45.6%	3.91% (2.37%, 5.48%)
		Female	39.01 (17.26, 68.37) → 106.49 (53.36, 182.17)	173%	3.51% (2.62%, 4.4%)	106.49 (53.36, 182.17) → 131.12 (48.21, 256.56)	23.1%	2.11% (1.22%, 3%)
		Male	108.48 (57.43, 174.67) → 250.28 (146.39, 376.38)	130.7%	2.75% (1.09%, 4.43%)	250.28 (146.39, 376.38) → 388.17 (225.49, 592.49)	55.1%	4.58% (2.78%, 6.41%)
	Italy	Both	201.18 (145.22, 278.87) → 576 (430.5, 793.23)	186.3%	3.86% (3.17%, 4.55%)	576 (430.5, 793.23) → 715.72 (513.4, 1001.35)	24.3%	2.57% (0.97%, 4.2%)
		Female	45.5 (30.93, 62.27) → 154.35 (93.07, 263.8)	239.2%	4.46% (3.59%, 5.33%)	154.35 (93.07, 263.8) → 193.24 (101.22, 330.58)	25.2%	2.67% (0.91%, 4.46%)
		Male	155.68 (108.39, 225.21) → 421.65 (311.84, 591.87)	170.8%	3.66% (3.02%, 4.3%)	421.65 (311.84, 591.87) → 522.47 (369.68, 726.83)	23.9%	2.54% (0.99%, 4.11%)
	Japan	Both	7.11 (5.68, 9.46) → 114.66 (74.69, 166.55)	1512.7%	15.08% (14.75%, 15.42%)	114.66 (74.69, 166.55) → 399.11 (227.96, 647.27)	248.1%	12.27% (11.94%, 12.59%)
		Female	2.43 (1.8, 3.39) → 35.21 (22.09, 55.35)	1349%	13.99% (13.82%, 14.17%)	35.21 (22.09, 55.35) → 130.69 (72.4, 212.38)	271.2%	12.81% (12.44%, 13.18%)
		Male	4.67 (3.73, 6.18) → 79.45 (53.02, 118.03)	1601.3%	15.61% (15.16%, 16.06%)	79.45 (53.02, 118.03) → 268.43 (152.77, 426.11)	237.9%	12.01% (11.68%, 12.34%)
	United Kingdom	Both	73.07 (58.28, 91.94) → 183.08 (130.42, 252.46)	150.6%	3.01% (1.63%, 4.4%)	183.08 (130.42, 252.46) → 456.23 (300.34, 648.66)	149.2%	8.6% (8.33%, 8.88%)
		Female	7.48 (5.5, 10.03) → 47.57 (30.78, 70.2)	536%	8.39% (7.46%, 9.33%)	47.57 (30.78, 70.2) → 137.97 (80.82, 211.86)	190%	10.27% (9.94%, 10.59%)
		Male	65.59 (52.69, 82.02) → 135.52 (97.24, 184.06)	106.6%	1.81% (0.22%, 3.43%)	135.52 (97.24, 184.06) → 318.26 (209.07, 447.23)	134.8%	7.96% (7.67%, 8.26%)
	United States of America	Both	1494.79 (1268.85, 1759.59) → 3294.61 (2568.82, 4242.03)	120.4%	2.79% (2.19%, 3.4%)	3294.61 (2568.82, 4242.03) → 6931.52 (4904.28, 9692.24)	110.4%	7.06% (6.84%, 7.28%)
		Female	450.65 (372.47, 534.07) → 843.35 (631.16, 1120.18)	87.1%	1.86% (1.13%, 2.59%)	843.35 (631.16, 1120.18) → 1424.64 (911.15, 2203.26)	68.9%	4.82% (4.42%, 5.21%)
		Male	1044.14 (873.51, 1247.02) → 2451.26 (1900.89, 3164.79)	134.8%	3.18% (2.56%, 3.81%)	2451.26 (1900.89, 3164.79) → 5506.89 (3903.86, 7675.43)	124.7%	7.73% (7.54%, 7.92%)

## 4 Discussion

This study, based on the GBD database, systematically delineated the long-term trends in incidence, mortality, prevalence, and comprehensive disease burden of HIV/AIDS and its related subtypes among individuals aged ≥75 years in G7 countries from 1990 to 2021, providing new evidence for understanding the epidemiological transition of older adult HIV-infected populations. Overall, ASIR and ASMR showed sustained declines in most countries, reflecting the long-term effectiveness of widespread ART implementation, improved early diagnostic systems, and enhanced long-term follow-up management. However, prevalence and DALYs exhibited upward trends in some countries, suggesting that HIV/AIDS among older adults is gradually transitioning from an acute fatal disease to a chronic condition characterized by long-term immune abnormalities, multisystem damage, and chronic health burden. This asymmetric pattern of “declining incidence and mortality with increasing survival and disability” aligns closely with the HIV epidemiological transition theory proposed in recent years and echoes the growing attention to chronic burden among older adult HIV-infected individuals.

The compounding effect of population aging and extended survival among HIV-infected individuals provides an important context for explaining these trends. ART significantly reduces viral load and improves immune function, enabling an increasing number of early-infected individuals to reach advanced ages, forming what has been termed “aging HIV cohorts.” However, long-term HIV infection accompanied by chronic inflammatory states (inflammaging) and sustained immune activation (11, 12) can accelerate immunosenescence (13, 14), leading to CD4 + T cell functional decline, restricted B cell activation, and reduced natural killer cell activity. This mechanism not only increases the risk of non-AIDS-related diseases (such as cardiovascular disease, metabolic syndrome, osteoporosis, cognitive impairment, and malignancies) but also manifests as continuous accumulation of disability burden in DALYs through the superimposed effects of chronic inflammatory networks and immune function decline. Therefore, even when incidence and mortality are effectively controlled, the overall disease burden among older adult HIV-infected individuals may continue to rise.

Inter-country differences further reveal the complexity of HIV/AIDS epidemiological characteristics in older adults. While the United States and several European countries experienced significant declines in incidence and mortality, prevalence and DALYs remained at relatively high levels, likely reflecting their early ART adoption, large surviving population base, and mature follow-up systems. In contrast, Japan showed sustained increases in incidence, mortality, and DALYs, presenting a distinctive “exception pattern” that may be associated with rapid population aging, insufficient HIV screening coverage, and inadequate attention to prevention strategies targeting older adult populations in terms of social awareness (15). Additionally, initial diagnosis in older adult patients often occurs at late stages with significant immune compromise, further exacerbating disease burden (15). These differences suggest that beyond healthcare accessibility, social behavioral factors, healthcare service utilization, and delayed diagnosis among older adults may all serve as important modulators of disease burden.

Sex differences were also evident in HIV/AIDS burden among older adults. The study revealed that overall burden in males aged ≥75 years exceeded that in females, consistent with higher HIV infection risk and lower healthcare service utilization among males in most countries. However, the sex gap narrowed at very advanced ages in some countries, possibly because higher mid-life mortality among male HIV-infected individuals reduces their proportion reaching very advanced ages, while females gradually accumulate at advanced ages due to overall survival advantages. Furthermore, females are more prone to multiple chronic comorbidities in old age, potentially increasing their relative contribution to prevalence and DALYs (16). This phenomenon suggests that sex differences in disease progression, comorbidity burden, and long-term health outcomes should be fully considered in the management of older adult HIV populations.

From a disease subtype perspective, the disease spectrum of HIV/AIDS is undergoing structural transformation. Incidence, mortality, and DALYs for HIV/AIDS with drug-susceptible tuberculosis continued to decline in G7 countries, while burden related to multidrug-resistant and extensively drug-resistant tuberculosis remained at extremely low levels over the long term, reflecting the effectiveness of coordinated HIV and tuberculosis control strategies. Conversely, other diseases resulting from HIV/AIDS have gradually become the primary source of DALYs, indicating that non-communicable chronic diseases are replacing opportunistic infections as the core drivers of health loss among older adults. This transition involves not only immunological mechanisms but is also closely related to long-term ART-associated metabolic complications (17, 18), chronic inflammatory states, declining organ function, and multimorbidity, emphasizing that management of older adult HIV-infected individuals needs to expand from mere viral suppression to comprehensive chronic disease management, multidisciplinary collaboration, and functional maintenance.

This study has important implications for public health policy and clinical practice. First, older adult populations should not be excluded from HIV prevention, screening, and health education; in particular, awareness of sexual behavior risks in older adults and early diagnostic coverage need to be enhanced (19, 20). Second, disease burden assessment should move beyond traditional incidence and mortality indicators to incorporate prevalence, DALYs, and chronic comorbidity burden into routine surveillance frameworks to comprehensively reflect long-term health loss. Third, HIV management strategies need to transition from a single model centered on viral suppression to a comprehensive management model that addresses geriatric syndromes, chronic comorbidities, cognitive function, and quality of life, including multidisciplinary team collaboration, cardiovascular and metabolic risk management, bone health maintenance, and cancer screening.

Although this study is based on high-quality GBD data, limitations should be noted. GBD estimates rely on multi-source data modeling, and HIV cases among older adult populations in some countries may be underestimated. Additionally, this study represents a macro-level ecological analysis and cannot explore individual-level behavioral, socioeconomic factors, or treatment adherence differences in depth. Additionally, this study focused exclusively on individuals aged ≥75 years without direct comparison to younger or middle-aged populations. Given the higher burden of comorbidities and competing mortality risks in older adults, observed trends in ASMR and ASPR may be influenced by non-HIV-related causes of death, potentially affecting the interpretation and comparability of age-specific disease burden patterns. Nevertheless, this study provides important global comparative evidence for understanding the long-term changes, mechanistic basis, and public health significance of HIV/AIDS burden among older adults, offering scientific evidence for future policy development, clinical management, and healthcare resource allocation targeting older adult HIV-infected individuals.

## Data Availability

Publicly available datasets were analyzed in this study. This data can be found at: https://ghdx.healthdata.org/gbd-2021.
